# Navigating cross-border institutional complexity: A review and assessment of multinational nonmarket strategy research

**DOI:** 10.1057/s41267-021-00438-x

**Published:** 2021-06-21

**Authors:** Pei Sun, Jonathan P. Doh, Tazeeb Rajwani, Donald Siegel

**Affiliations:** 1 Alliance Manchester Business School, University of Manchester, Manchester, UK; 2 Villanova School of Business, Villanova University, Villanova, USA; 3 Surrey Business School, University of Surrey, Guildford, UK; 4 School of Public Affairs, Arizona State University, Tempe, USA

**Keywords:** corporate political activity, strategic corporate social responsibility, nonmarket strategy, multinational enterprise

## Abstract

Multinational enterprises are deeply engaged in nonmarket strategy (NMS), including both corporate political activity (CPA) and strategic corporate social responsibility (SCSR). In this review, we document the multinational NMS research according to contributions’ theme, method, context, theory, and level of analysis. We then develop an institutional multiplicity framework to organize our analysis of this large and fragmented body of literature. In so doing, we identify the most impactful contributions within three major themes – multinational CPA, multinational SCSR, and the integration of CPA and SCSR – and their respective subthemes, and call attention to limitations in the extant research. We also highlight promising avenues for future research, including expanding the scope of NMS to incorporate microfoundations research, integrating macrolevel scholarship on global institutions, placing greater attention on the interaction between CPA and SCSR, and incorporating multi-actor global issues and movements. Our review underscores the growing importance and missed opportunities of NMS research in the international business field.

**Electronic supplementary material:**

The online version of this article (10.1057/s41267-021-00438-x) contains supplementary material, which is available to authorized users.

## Resume

Les entreprises multinationales s’engagent profondément dans la stratégie hors-marché (NMS - Nonmarket Strategy), y compris à la fois l'activité politique des entreprises (CPA - Corporate Political Activity) et la responsabilité sociale stratégique des entreprises (SCSR - Strategic Corporate Social Responsibility). Dans cette revue de littérature, nous structurons la recherche sur la NMS des entreprises multinationales en fonction du thème, de la méthode, du contexte, de la théorie et du niveau d’analyse des contributions. Nous développons ensuite un cadre conceptuel fondé sur la multiplicité institutionnelle afin d’organiser notre analyse de ce corpus de recherche, aussi vaste que fragmenté. Ce faisant, nous identifions les contributions les plus influentes dans trois grands thèmes - CPA multinationale, SCSR multinationale et l’intégration entre CPA et SCSR -, ainsi que dans leurs sous-thèmes respectifs. Nous attirons par ailleurs l'attention sur les limites de la recherche existante. Nous soulignons également des pistes prometteuses pour de futures recherches, y compris l'élargissement de la portée de la NMS en vue d’incorporer la recherche sur les microfondations, l'intégration de la recherche au niveau macro sur les institutions globales, l’accord d’une plus grande attention à l'interaction entre CPA et SCSR, ainsi que l’incorporation des mouvements et des défis mondiaux multi-acteurs. Notre revue de littérature met en lumière l'importance croissante et les opportunités manquées de la recherche sur NMS dans le champ des affaires internationales.

## Resumen

Las empresas multinacionales están profundamente comprometidas en estrategias no comerciales (NMS por sus siglas en inglés), incluyendo tanto la actividad política corporativa (CPA por sus iniciales en inglés) y la responsabilidad social corporativa estratégica (SCSR por sus iniciales en inglés). En esta revisión, documentamos la investigación sobre estrategias no comerciales multinacionales de acuerdo con el tema, el método, el contexto, la teoría, y el nivel de análisis de las contribuciones. Luego, desarrollamos un marco de multiplicidad institucional para organizar nuestro análisis de este amplio y fragmentado conjunto de publicaciones. Al hacerlo, identificamos las contribuciones con más impacto en los tres temas más grandes -la actividad política corporativa multinacional, la responsabilidad social corporativa estratégica multinacional, y la integración de la actividad política corporativa y la responsabilidad social corporativa estratégica- y sus respectivos sub-temas, y llamamos la atención a las limitaciones de la investigación existente. También resaltamos avenidas promisoras para la investigación futura, incluyendo la ampliación del alcance de las estrategias no comerciales para incorporar la investigación de los microfundamentos, al integrar el conocimiento académico a nivel macro sobre las instituciones globales, poniendo la mayor atención en la interacción entre la actividad política corporativa y la responsabilidad social corporativa estratégica, e incorporando los asuntos y movimientos globales multi-actor. Nuestra revisión subraya la creciente importancia y las oportunidades perdidas de la investigación sobre estrategias no comerciales en el campo de los negocios internacionales.

## Resumo

Empresas multinacionais estão profundamente engajadas na estratégia de não mercado (NMS), incluindo tanto a atividade política corporativa (CPA) quanto a responsabilidade social corporativa estratégica (SCSR). Nesta revisão, documentamos a pesquisa sobre NMS multinacional de acordo com o tema, método, contexto, teoria e nível de análise das contribuições. Em seguida, desenvolvemos um modelo de multiplicidade institucional para organizar nossa análise desse grande e fragmentado corpo de literatura. Ao fazer isso, identificamos as contribuições mais impactantes dentro de três temas principais - CPA multinacional, SCSR multinacional e a integração de CPA com SCSR - e seus respectivos subtemas, e chamamos a atenção para limitações da pesquisa existente. Também destacamos caminhos promissores para pesquisas futuras, incluindo a expansão do escopo de NMS para incorporar pesquisa de micro fundamentos, integração estudos acadêmicos em nível macro em instituições globais, dando maior atenção à interação entre CPA e SCSR e incorporando questões e movimentos globais de múltiplos atores. Nossa análise ressalta a importância crescente e oportunidades perdidas na pesquisa de NMS no campo de negócios internacionais (IB).

## 摘要

跨国企业深深地参与到非市场战略 (NMS)行动中, 这既包括企业政治活动 (CPA) 和战略性企业社会责任行动 (SCSR)。在这篇综述文章中, 我们根据作品的主题,、方法、情境、理论和分析层面, 对跨国企业非市场战略活动的研究进行了详细描述与解读。 具体而言, 我们建立了一个制度多元性的框架来组织我们对这一庞大而零散的文献的分析。 通过这种做法, 我们在三个主要主题 (跨国CPA, 跨国SCSR, 和CPA与SCSR的整合) 及各自的子主题中识别、确定了最具影响力的研究贡献, 并呼吁读者关注现有研究中的局限性。我们还重点介绍了该领域未来研究的有前途的方向, 包括扩大非市场战略研究范围以纳入微观基础研究, 整合有关全球制度的宏观研究, 更加关注CPA和SCSR之间的互动, 以及研究中纳入有多个参与者的全球议题和社会运动。我们的综述文章强调了非市场战略研究在国际商务领域中日益重要的地位和亟需把握的机会。

## INTRODUCTION

Nonmarket strategy (NMS) is a firm’s concerted action to improve its competitive position and performance by actively managing the institutional or societal contexts of business competition in which it operates (Mellahi, Frynas, Sun, & Siegel, 2016: 144). It is comprised of two interrelated components: corporate political activity (CPA) and strategic corporate social responsibility (SCSR), and unfolds at multiple levels of analysis. Multinational enterprises (MNEs) are engaged in continuous interactions with sociopolitical stakeholders in their home, host, and supranational nonmarket environments, and are thus deeply involved in NMS. Those differing social, political, and institutional contexts require MNEs to devise nonmarket strategies to navigate such cross-border complexity. Despite this reality, research on multinational NMS was still considered “embryonic” in the mid-2000s (Rodriguez, Siegel, Hillman, & Eden, 2006).

The 2010s witnessed burgeoning research on NMS, leading to important assessments from various perspectives (Dorobantu, Kaul, & Belner, 2017; Marquis & Raynard, 2015; Mellahi et al., 2016; Tihanyi et al., 2019). Concurrently, there has been a noticeable increase in international business (IB) scholarship addressing NMS as a critical component of global strategy (Boddewyn & Doh, 2011; Cuervo-Cazurra, Inkpen, Musacchio, & Ramaswamy, 2014; Doh, McGuire, & Ozaki, 2015; Doh, Rodrigues, Saka-Helmhout, & Makhija, 2017; Kobrin, 2015). However, we still lack a systematic assessment of NMS research *with reference to the IB field in general and to the MNE in particular*. This omission motivates us to conduct the present review. Of note, compared with domestic firms, MNEs crossing borders face more diverse and possibly conflicting sets of institutional contexts and stakeholders in their NMS formulation and execution (Kostova & Zaheer, 1999; Kostova, Roth, & Dacin, 2008). Hence, evaluating the achievements and limitations of multinational NMS literature represents an important avenue for advancing IB and strategy research.

To this end, we integrate the fragmented multinational NMS research by taking stock of scholarship primarily during 2000–2020, and developing a conceptual framework to organize our ensuing review of how MNEs navigate cross-border institutional complexity via their nonmarket strategies (Elsbach & van Knippenberg, 2020). In so doing, we clarify the status, contribution, and future directions of NMS research in the IB field. Specifically, we address three components of NMS research: CPA, SCSR, and the integration of CPA and SCSR in the international context. We also seek to stimulate a dialogue within the broader IB scholarly community about how we can collectively better understand multinational nonmarket strategies.

Given that NMS is an area that draws from several disciplinary traditions and is concerned with multiple levels of analysis, we hope to enhance and stimulate scholarship among three communities. First, we seek to contribute insights to IB scholars who are interested in how MNEs combine market and nonmarket strategies to create and sustain competitive advantages globally. IB research has a strong tradition of studying how internationalization activities (e.g., location choice, entry mode, and partner selection) are shaped by nonmarket institutions such as political risk and corrupt environments (Sartor & Beamish, 2018; Vaaler, 2008). While recognizing the importance of this research, we instead focus on studies that examine MNEs’ *direct* engagement with – and actions focused toward – sociopolitical stakeholders at the individual and organizational levels.^1^ Given the mounting global sociopolitical challenges facing MNEs, our review helps address big questions and grand challenges in IB research (Buckley, Doh, & Benischke, 2017).

Second, we contribute to strategy scholarship on the antecedents and effectiveness of NMS as it relates to factors intrinsic to international environments. While domestic firms in developed and emerging economies alike have been dealing with challenging nonmarket environments and demanding sociopolitical stakeholders, our review offers fresh insights into NMS research. The sheer complexity faced by MNEs when they engage with home-country, host-country, and supranational sociopolitical actors leads them to develop a rich strategy toolkit for managing legitimacy concerns raised by and resource exchanges with external environments and stakeholders (e.g., De Villa, Rajwani, Lawton, & Mellahi, 2019; Marano, Tashman, & Kostova, 2017; Sun, Mellahi, & Thun, 2010; Zhang & Luo, 2013). Moreover, our review adds to a growing stream of NMS research that examines the potential for integrating different elements of NMS by combining CPA and SCSR, demonstrating how MNEs manage the complementarity and tensions between these two components (Darendeli & Hill, 2016; Montiel, Husted, & Christmann, 2012; Stevens, Xie, & Peng, 2016).

Finally, we speak to a broader community of scholars from economics, political science, sociology, and management studies, who have contributed to the emerging NMS field but have yet to realize its dynamism in the international context. We believe the cross-fertilization is a two-way process: Our review has incorporated insights from a number of important studies published in these disciplines (e.g., Gawande, Krishna, & Robbins, 2006; Hoang, 2018; Khan, Lew, & Park, 2015). However, we also hope our review can stimulate further interests for social scientists to enrich our understanding of this topic from complementary perspectives.

In this article, we first provide a detailed description of our review methodology and report our results on the distribution of articles by journal type/category, major themes, research methodology, country/region identity, and theoretical perspectives. Next, we identify three broad areas of NMS research – CPA, SCSR, and the integration of CPA and SCSR – and develop an analytical framework highlighting the ways in which MNEs employ these nonmarket tactics to address the institutional multiplicity facing them. Further, we use a citation analysis to motivate discussions about the most prominent contributions, research gaps, and areas for additional research *within* each of the topical areas. Finally, we provide concluding thoughts and insights as to productive *overall* future research directions.

## METHODOLOGY

We focus on research findings reported in the last 21 years (2000–2020) that explicitly address the antecedents, organization, effectiveness, and consequences of multinational NMS. Following typical journal selection processes in top journal review articles (e.g., Mellahi et al., 2016; Meyer, Li, & Schotter, 2020), we include general management journals (*Academy of Management Journal*, *Academy of Management Review*, *Administrative Science Quarterly*, *Journal of Management*, *Journal of Management Studies*, *Management Science*, *Organization Science*, *Organization Studies*, and *Strategic Management Journal*), IB journals (*Global Strategy Journal*, *International Business Review*, *Journal of International Business Studies*, *Journal of International Management*, *Journal of World Business*, and *Management International Review*), and journals focusing on the interface between business and society (*Business Ethics Quarterly*, *Business & Politics*, *Business & Society*, and *Journal of Business Ethics*). Finally, realizing the cross-disciplinary nature of our review topic, we conducted an extensive search of relevant articles in a total of 44 leading journals in economics, finance, marketing, political science, and sociology. Due to space limitations, the full list of the journals is not reported here but available upon request.

We adopted a multistep search methodology. First, we searched articles with phrases like “corporate political strategy,” “corporate political activity,” “corporate social responsibility,” “nonmarket strategy,” “nonmarket environment,” “multinational enterprise,” and “multinational companies” in the title, abstract, and keywords section using the EBSCO database (Doh & Lucea, 2013). Specifically, our search used the following search strings:

*CSR* = (Corporate social responsibility* OR CSR OR social strateg* OR stakeholder OR corporate citizen* OR ethic* OR sustainab* OR climate OR corporate philanthropy OR donation OR corporate giv* OR corporate citizen* OR NGO OR climate*)

*CPA* = (Nonmarket strateg* OR corporate political strateg* OR political connection* OR political tie* OR corporate political activit* OR lobby* OR campaign contribution* OR political risk)

*Internationalization* = (International OR subsidiar* OR MNE OR MNC OR TNC OR foreign* OR cross-border OR multinational OR transnational OR global OR home OR host)

Second, we recognized that NMS is often embedded in more broadly constructed studies. For example, corruption^2^ and philanthropy by definition involve strategic interactions among firms, governments, and civil society actors, but are rarely presented under the rubric of “nonmarket” or “political” strategy. As such, we broadened our search to incorporate a wider range of issues that characterize the nonmarket environment and nonmarket activities, even if not explicitly labeled “nonmarket” or “strategy.” Specifically, two members of the author team manually checked each journal issue to identify relevant nonmarket research papers that did not emerge from the above keyword search.

Third, we followed the tradition of the *Journal of International Business Studies* (*JIBS*) and other top IB journals by including only articles that explicitly addressed the “cross-border” or comparative dimension of corporate nonmarket activities. That is, we excluded all single-country articles without a cross-border element and those simply using large cross-national samples to study the behavior of domestic firms.

Finally, as noted in the Introduction, we excluded studies that only examine firms’ business responses/adaptations to home-/host-country nonmarket environments. In other words, we require the presence of direct nonmarket activities and factors at the individual and organizational levels, such as government ownership, managerial political ties, lobbying, sustainability reporting, and corporate philanthropy. These variables need to serve as dependent, independent, or moderating/mediating variables (rather than control variables) in the articles selected for our review. On the basis of these criteria, our search resulted in a total of 367 articles (381 including those prior to 2000).

We coded the articles across a range of dimensions by carefully reading each. One author took the primary coding responsibility, while two other authors cross-checked the coding to ensure reliability. When discrepancies arose, we discussed and fine-tuned the coding results. First, we coded the primary topics of the NMS research, namely CPA, SCSR, and CPA & SCSR. The “CPA & SCSR” category refers to articles that covered CPA- and CSR-related variables simultaneously in theory/hypotheses development and were then coded for subthemes. In CPA articles, we found subthemes such as SOE internationalization strategy and MNE–host government bargaining; regarding SCSR articles, we coded for topics such as environmental issues and CSR reporting.

Second, we coded for the primary theories and the research methodology used in the studies. The theories ranged from broad social science theories, such as institutional theory, to narrower ones, such as the political risk literature. Regarding methodology, we first distinguished conceptual articles from empirical ones. The former contained theory development works, literature reviews, perspective articles, and overviews of journal special issues. The latter were further classified as quantitative, qualitative, and mixed method articles.

To delineate cross-border institutional multiplicity, which is the defining feature of this literature, we coded for the types/levels of home- and host-country sociopolitical institutions with which the articles have engaged in developing their research questions or hypotheses. We also checked if supranational institutions (such as WTO, diplomatic relations, and bilateral treaties) were incorporated into the analytical framework of the selected articles. We only coded variables where there was an explicit application in the main research questions and models, excluding information if only used as control variables. As such, we ensured that the articles really engaged with them in dependent, independent, or moderating/mediating variables.

We then undertook several analyses, relying in part on citations to determine the relative influence of the broad areas (CPA, SCSR, CPA & SCSR) and the specific topics within those broad areas. We used this analysis to identify both major contribution streams and gaps within each area. Before focusing on our broad sample that begins in 2000, we identified the most highly cited articles from before 2000 that could be broadly construed as “NMS” even though the term “nonmarket” was rarely used then.

## THE HISTORICAL ANTECEDENTS OF NONMARKET STRATEGY RESEARCH IN IB

Although nonmarket strategy is a relatively recent addition to IB research, some of the foundational concepts have a long history in IB (Grosse, 2005). For instance, two pioneers of the IB field – Hymer (1960/1976) and Vernon (1971) – dealt with issues related to international business–government relationships. In this regard, several influential books published between the 1970s and the 1990s reflected the increasing tensions between MNEs and host-country governments, especially in the developing world, and the mechanisms through which those tensions played out. These included Vernon’s *Sovereignty at Bay* (1971), which introduced the notion that MNE bargaining power with host-governments “obsolesces” over time, Barnet and Muller’s *Global Reach* (1974), which explored the range of impacts MNEs have on governments, taxpayers, workers, and business, and Moran’s (1974) study of MNE–host interdependencies in the context of copper in Chile. These contributions, in turn, led to additional insights into and empirical tests of the dynamics of negotiation and bargaining between MNEs and host governments (Fagre & Wells, 1982; Kobrin, 1987), and how these interdependencies among MNEs and hosts evolve over time (Stopford & Strange, 1991; Vachani, 1995). Stopford and Strange (1991) showed how structural changes in the global economy had incentivized host governments to seek cooperation from MNEs, while balancing political, economic, and societal imperatives, leading to differing bargaining relationships in various countries and sectors that evolved as contextual conditions changed.

Concurrent with these contributions was a literature stream in the broad area of ethics and corporate responsibility, that proposed that some ethical behaviors and “norms” were universal while others were context-specific. Donaldson and Dunfee (1994, 1999) term the former “hypernorms” and the latter “local” norms. This stream helped set the stage for later IB research that suggested that some NMS actions in the realm of CSR could be carried from one country to another, while others had to adapt to local pressures (e.g., Tashman, Marano, & Kostova, 2019). Although Baron did not develop the “nonmarket” construct until the mid-1990s (Baron, 1995a, 1995b), and that term was not picked by IB for some time thereafter, these earlier contributions provided the antecedents for NMS-related research that we discuss below.

To capture these early insights, we undertook a supplemental analysis to identify important IB/NMS publications pre-2000, using a similar Boolean search protocol as for our main analysis. We took a somewhat broader approach than in our more targeted analysis, given the relative paucity of studies and our goal of identifying the relevant foundational research for later studies. Not surprisingly, we found relatively few scholarly publications in this domain pre-2000. Online Appendix Table A lists a total of 14 publications that generated more than 60 total citations by mid-February 2021, as reflected in the Web of Science database. They fall mostly into two categories – research on political risk, and research on international business–government relations/MNC–host government bargaining – and include such leading IB scholars as Jean Boddewyn, Thomas Brewer, Stephen Kobrin, Stephanie Lenway, and Thomas Murtha. Some of this early research took the form of books and/or practitioner-oriented articles. One classic contribution that continues to influence the NMS literature was Ray Vernon’s *Sovereignty at Bay* (1971), which introduced the notion of the obsolescing bargain to capture the changing power and influence between MNEs and host countries.

The earliest articles (Fitzpatrick, 1983; Kobrin, 1979; Robock, 1971) are perspective/review papers on the practicalities of political risk. These likely reflect the contexts at that time when host governments, especially those in Latin America, engaged in various forms of expropriation of foreign businesses. As the literature evolved in the 1980s and 1990s, there emerged more sophisticated constructs dealing with business–government relations in the global environment (Boddewyn & Brewer, 1994; Hillman & Keim, 1995), CPA in response to international trade issues (Schuler, 1996), and stakeholder management in international joint ventures (Brouthers & Bamossy, 1997).

Boddewyn and Brewer (1994) stand out as one of the most influential contributions from this era. It is one of the first IB articles to acknowledge the explicit and active role of MNEs in international political activity. It develops a comprehensive model to understand when and how MNEs engaged in political behavior, and how the political context of various jurisdictions constrains or enables those efforts. In effect, this conceptual work reorients the conventional political risk and MNE–host government relationship research toward a firm-specific, managerial focus, contending that “the analysis of IB political behavior requires…consideration of what may be called *organizational strategies* regarding the effective development and use of actions, structures, and processes toward the nonmarket environment” (p. 137, italics in original). This is one of the most highly cited of these early contributions, and lays the foundation for much of the later international CPA stream.

It is notable that social responsibility, sustainability, and related topics were not widely reflected in IB research prior to 2000, although, as noted above, there was a related stream in management research on comparative ethics. While allied disciplines had already ventured into this area, IB was late in adopting a broader, more integrative perspective regarding the MNE and its social and environmental responsibilities. Building on the work of Penrose and Barney, Rugman and Verbeke (1998) were among the first to explore the development of corporate “green capabilities.” This resource-based view of green capabilities in response to national and international environmental regulations provided the basis for a number of later contributions in the realm of multinational SCSR research. In short, there were important – but limited – conceptual developments in the 1990s to situate MNE activities in the sociopolitical arena and discuss proactive strategic responses. However, in the twenty-first century, empirical inquiries of MNE nonmarket activities began to flourish in the IB field. Hence, the “embryonic” assessment by Rodriguez et al. (2006) in their introduction to the first *JIBS* special issue on this topic.

## RESULTS OF LITERATURE SEARCH

In this section, we provide descriptive results of our review by the major data classification categories we employed. A number of important observations have emerged from our analysis of these results.

### Results by Journal and Year

Online Appendix Table B presents a breakdown of the 367 articles in terms of publication year, journal type, and nonmarket activities investigated. As shown in the table, a total of 273 articles were published between 2011 and 2020, compared to just 94 articles in the first decade of the century. Interestingly, the increase is largely driven by the growing popularity of the NMS topic in IB journals, with that group contributing 60% of total publications. More than 80% of multinational NMS articles were published in IB journals after 2009, underscoring how NMS research has become increasingly crucial in IB scholarship.

It is noteworthy that the 367 papers identified by our literature search have a rather limited overlap with recent reviews of general NMS. In the case of Mellahi et al. (2016), only 18 of the 214 articles they reviewed are also included in our dataset. Dorobantu et al. (2017) and Marquis and Raynard (2015) do not formally report their collections of research articles reviewed; however, judged by their reference lists, our overlap is 12 and 5 articles, respectively. These comparisons suggest that extant NMS research in heavily weighted toward the single-country setting, and make a strong case for assessing multinational NMS research.

### Results by Theme

Online Appendix Table C presents data on the theme by journal, showing a balance in coverage of the two primary topics: a total of 173 articles focused primarily on CPA, while 141 articles focused primarily on SCSR. In addition, 53 articles addressed both CPA and SCSR. Examining themes from the perspective of journal type, we find that IB journals traditionally published more CPA research, while nonmarket specialist journals appear to have a particular focus on SCSR. IB journals also published more articles on the integration between CPA and SCSR than did the other two types of journals. In terms of individual journals, *JIBS* is the leading outlet for multinational NMS research, as its research articles account for 20% of the total.

### Results by Method

Online Appendix Table D presents data on the research methods by journal. Of the 367 articles, 63 (17%) were conceptual, including theory development, review/perspective articles, and overview of journal special issues. The majority were empirical, with 205 articles (56%) employing quantitative methods and 95 (26%) using qualitative methods. Only four articles (1%) combined quantitative and qualitative methods. Regarding conceptual articles, IB journals published about two-thirds of the total, with many of these taking the form of literature reviews, perspective articles, and special issue introductions. However, with the exception of two articles published in *Academy of Management Review* in the mid-2000s, theory development articles on the multinational dimension of corporate nonmarket activity were mostly absent in general management journals until 2019 (Asmussen & Fosfuri, 2019).

With respect to empirical work, quantitative research is dominant in both general management and IB journals, whereas a more balanced quantitative–qualitative methodology distribution is found in nonmarket specialist journals. *JIBS* alone published more than one-quarter of the quantitative studies (54 out of 205). By contrast, *JIBS* published only six qualitative articles and one mixed method article over the same period. In comparison with other journals, *Journal of World Business* and *Journal of Business Ethics* are important outlets for qualitative articles in this area.

### Results by Country/Region Focus

Finally, Online Appendix Table E reports the research contexts of the articles by listing the countries/regions the articles focus on for home and host dimensions. China ranks first, suggesting its involvement in more than one-quarter of empirical studies of multinational NMS. Further, the Chinese context is evenly distributed between home- and host-country dimensions, which include both Chinese firms going global and MNEs doing business in China. In the case of developed economies, the United States, Western Europe, and Japan rank second, third, and seventh, respectively, with the majority of studies focusing on the home-country dimension. Conversely, emerging and developing economies like Latin America, Africa, and India are popular host-country targets.

### Results by Theory

Table 1 reports the primary theoretical perspectives that this body of literature draws upon. For the sake of brevity, we only list theories that have been employed by more than ten articles in our dataset. Several notable patterns must be discussed. First, the primary theories identified in our review are similar to those in earlier reviews of general nonmarket strategy (Mellahi et al., 2016), except that social network/embedded perspective and transaction cost economics (TCE) are additional primary theoretical angles in the multinational NMS literature.

**Table 1:** Primary theoretical perspectives and major themes

	CPA	SCSR	CPA & SCSR	Total number
Agency theory	11	2	2	15
Institutional theory and its variants (e.g., new institutional economics, institution-based view, neo-institutional theory, the legitimacy perspective, and the comparative capitalism literature)	45	59	21	125
Resource-based view (RBV; including the dynamic capabilities variant)	20	13	4	37
Resource dependency theory (RDT)	24	2	4	30
Social network/embeddedness theory	8	3	6	17
Stakeholder theory	1	23	8	32
Transaction cost economics (TCE)	9	5	2	16
IB-based perspectives (e.g., ownership, location, internalization paradigm, internalization theory, internationalization process/stage theories, and the liability of foreignness literature)	25	6	2	33

A total of 323 out of the 367 articles stated the theories/perspectives applied in their research. Here, we only list theories that have been used by more than 10 articles in our dataset.

More importantly, institutional theory and its variants are by far the most dominant in multinational NMS research. This pattern is different from the fairly even distribution of theory application revealed by Mallahi and colleagues (2016) in their review of general NMS research. We believe this is because the institutional perspective is intrinsic to the international aims of this strand of literature: understanding the challenges and opportunities involved in navigating home, host, and supranational institutions across the globe.

Further, none of the individual “mid-range” theoretical perspectives that originate from the conventional IB literature (e.g., internalization theory) reach the ten-article threshold in our dataset. Taken together, this group of conventional IB-based theories was used in 33 articles. This suggests that penetration of “mainstream” NMS research in the IB context is more extensive than the other way around. Finally, in terms of theories that are disproportionately used to inform one of the two main themes of NMS research, resource dependence theory (RDT) ranks second in theoretical popularity in the subfield of CPA research, while stakeholder theory does so in SCSR research as well as in the integrated CPA & SCSR research.

## CHARTING THE INFLUENCE OF NONMARKET STRATEGY RESEARCH: ANALYTICAL FRAMEWORK, CITATION ANALYSES, AND CRITICAL ASSESSMENT

### Analytical Framework

**Figure 1:**
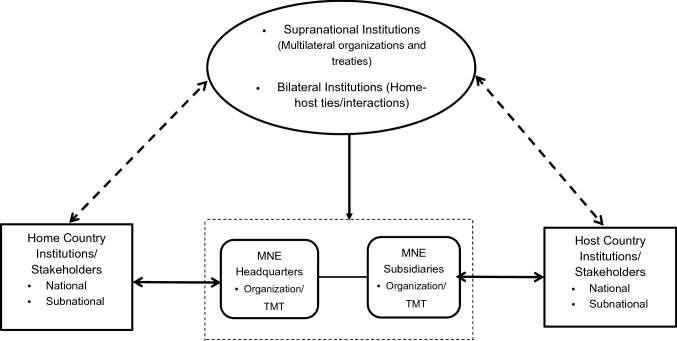
Institutional multiplicity and MNE nonmarket strategy. *Solid lines *denote interactions between the focal MNE and the institutional arrangements and stakeholder groups, while *dotted lines *suggest the interactions between the multiple sets of institutions and stakeholder groups.

We here focus on the unique challenges faced by MNEs in navigating cross-border institutional complexity. Our analytical framework of institutional multiplicity builds on and extends insights from Ramamurti (2001), who developed a two-tiered model of MNE bargaining that reflects the roles of home, host governments and multilateral institutions, and Kostova and Zaheer (1999: 70), who emphasize the reality that “MNEs face multiple country institutional environments”, and that this “multiplicity and variety…clearly differentiate MNEs from domestic firms.” As Kostova and Zaheer note, MNEs face multiple institutional challenges: home-based, host-based, supranational, and the interaction between these. In response, MNEs can develop nonmarket strategies to address such challenges. For example, to overcome the liability of foreignness, MNEs and their subsidiaries conduct CPA and SCSR to engage with host-country sociopolitical institutions and stakeholders, while MNE headquarters manage similar home-country interactions. Further, MNEs’ strategic actions are subject to supranational institutional pressures and home-host-country relationships. In addition, MNE nonmarket strategies must address multiple and often conflicting institutional pressures from home, host, and supranational levels. Finally, within the MNE, NMS formulation and execution are regulated by headquarter–subsidiary relationships and influenced by the characteristics of its top management teams (TMTs) in both the headquarters and the subsidiaries (Meyer, Mudambi, & Narula, 2011; Meyer et al., 2020). It is through this lens, as depicted in Figure 1, that we analyze the contributions of the multinational NMS literature.

In view of this multilevel institutional analysis, Table 2 presents how the various dimensions of home, host, and supranational institutions have been covered by the field. Specifically, we found 110 primarily “host-focused” studies and 52 primarily “home-focused” studies that examined national-level institutions only. In addition, 23 studies investigated both national and subnational host-country institutions and six studies combined both levels of home-country institutions. We found subnational institutions clearly understudied: only four articles concentrated on subnational host-country institutions, while no study concentrated on subnational home-country institutions. In multilevel studies, the most common approach concerns the combination of host- and home-country institutions, for a totals of 66 “home–host” studies. A total of 62 articles engaged in some way with supranational institutions in their conceptual/hypotheses development and/or empirical analysis. Finally, we identified very few studies that incorporated NMS headquarter–subsidiary relationships and individual agency decision-making, whether among the top management team or among employees overall. This is an issue we return to in the subsequent sections.

**Table 2:** Article distribution across the levels of institutional analysis and major themes

	CPA	SCSR	CPA & SCSR	Total number
Host-focused studies				
National level only	59	38	13	110
Subnational level only	0	2	2	4
National and subnational levels	12	5	6	23
Subtotal	71	45	21	137
Home-focused studies				
National level only	32	17	3	52
Subnational level only	0	0	0	0
National and subnational levels	6	0	0	6
Subtotal	38	17	3	58
Home–host studies	27	34	5	66
Supranational studies	31	17	14	62

There are 44 articles that do not seem to engage explicitly with any level of institutional analysis in our dataset. For instance, some research looks at the effect of firm-level social performance on the firm’s degree of internationalization. Some conceptual articles, such as literature reviews and special issue introductions, are not amenable to this classification either.

Below, we have leveraged citation analyses to identify the most influential themes in nonmarket strategy research across our three areas of focus, using the schema depicted in Figure 1. We drew on the Web of Science Citation database to generate total citations for each article in our sample. We applied a cutoff of 70 total citations (as of mid-February 2021) to determine which articles are included in the citation tables regarding CPA and SCSR, while applying 50 total citations as the cutoff for the younger stream of literature on CPA–SCSR integration. Further, our review also paid attention to relatively highly cited papers published during 2016–2020 that demonstrate strong early impact with less time to accumulate citations. It is worth noting that we do not limit our discussion exclusively to these highly cited works, and nor do we consider research significance as solely a function of these citations, as we also seek to identify underexplored areas and use them to build the case for future research opportunities.

### Multinational CPA Research

Per our selection criteria, Table 3 presents the top 10, and Online Appendix Table F identifies the complete list, of the most highly cited CPA articles. Considering these articles with others in the dataset, we identify four major themes: SOE internationalization strategy, MNE–host government relationships and bargaining, political risk/hazard management, and corporate political ties. We review this stream of research in line with our multilevel analytical framework. It is noteworthy that our review of the multinational CPA research is not organized on the basis of the conventional single-country CPA research template (Hillman & Hitt, 1999; Hillman, Keim, & Schuler, 2004), which consists of political action approach (relational vs transactional), participation level (individual vs collective), and strategy type (information, financial incentive, and constituency building strategies). Our reading of the IB-based CPA research suggests that MNEs typically combine different approaches and a host of political strategies to engage with host- and home-country political agencies and actors. Thus, we believe that organizing the discussion on the basis of topical areas rather than political strategy tactics may yield more informative insights.^3^

**Table 3:** Top 10 most highly cited articles on international CPA ranked by Web of Science citations

	Authors (year)	Article	Topic(s)	Type	Theory	Total cites	Cites/year
1	Delios and Henisz (2003)	Political hazards, experience, and sequential entry strategies: the international expansion of Japanese firms, 1980–1998	Political risk; foreign entry strategy	Empirical (Quantitative)	The stages model of Internationalization	378	21
2	Hitt, Bierman, Uhlenbruck, and Shimizu (2006)	The importance of resources in the internationalization of professional service firms: the good, the bad, and the ugly	Firm internationalization; political ties	Empirical (quantitative)	RBV	358	23.9
3	Cui and Jiang (2012)	State ownership effect on firms' FDI ownership decisions under institutional pressure: a study of Chinese outward-investing firms	SOE internationalization strategy	Empirical (quantitative)	Institutional theory and RDT	319	35.4
4	Holburn and Zelner (2010)	Political capabilities, policy risk, and international investment strategy: evidence from the global electric power generation industry	Political risk; location choice	Empirical (quantitative)	RBV and TCE	297	27
5	Wang, Hong, Kafouros, and Wright (2012)	Exploring the role of government involvement in outward FDI from emerging economies	SOE internationalization strategy	Empirical (quantitative)	Resource-based and institutional perspectives	271	30.1
6	Hillman and Wan (2005)	The determinants of MNE subsidiaries' political strategies: evidence of institutional duality	Subsidiary-level political strategies	Empirical (quantitative)	Institutional theory	211	13.3
7	Mezias (2002)	Identifying liabilities of foreignness and strategies to minimize their effects: the case of labor lawsuit judgments in the United States	Managing liabilities of foreignness	Empirical (quantitative)	FDI/liability of foreignness literature	211	11.1
8	Frynas, Mellahi, and Pigman (2006)	First mover advantages in international business and firm-specific political resources	MNE–host government relationship	Empirical (qualitative)	RDT and RBV	209	13.9
9	Siegel (2007)	Contingent political capital and international alliances: Evidence from South Korea	Political ties	Empirical (quantitative)	Social network theory	194	14.9
10	Cuervo-Cazurra et al. (2014)	Governments as owners: state-owned multinational companies	SOE internationalization	Conceptual (special issue editorial)	Agency theory, TCE, RBV, RDT, and institutional theory	187	26.7
10	Henisz and Zelner (2005)	Legitimacy, interest group pressures, and change in emergent institutions: the case of foreign investors and host country governments	MNE–host government relationship/bargaining	Conceptual	Institutional theory	187	11.7

The citation data were collected from the Web of Sciences in mid-February 2021.

*FDI* Foreign direct investment, *RBV* resource-based view, *RDT* resource dependency theory, *TCE* transaction cost economics.

#### SOE internationalization strategy

The 2010s witnessed a surge of research on the role of state ownership in shaping internationalization strategy and outcomes. This is due to increasing interest in understanding emerging market firm (EMF) internationalization, as home-country governments often have ownership involvements in these firms. This topic has attracted the academic spotlight and involved a critical mass of influential *JIBS* articles (Cui & Jiang, 2012; Wang et al., 2012), including a collection of special issue articles in 2014 (Cuervo-Cazurra et al., 2014).

First, *home-country political institutions* play a prominent role in driving the globalization of SOEs. The extant literature has developed an elaborate account of the interplay between home governments and focal firms via state ownership linkages. Compared with private MNEs, government-created advantages in the form of financial and political/policy resources can lead SOEs to engage in more internationalization and riskier overseas projects (Ramamurti & Hillemann, 2018). Further, research also develops a disaggregate understanding of the home political institutions. For instance, Wang et al. (2012) distinguish two dimensions of political influences – state ownership and government affiliation level – and examine their differential effects on firms’ FDI strategy. Li and colleagues (2014) develop a central–local taxonomy to explain the variance in SOE internationalization motives and strategies. The literature also notes the dark side of home political institutions, highlighting the political nature of SOEs by studying the lack of transparency in these organizations and their FDI projects. Cannizzaro and Weiner (2018) suggest a negative association between state ownership and FDI-project transparency, especially when firms are from countries with weak institutional quality, while Li, Li, and Wang (2019) confirm that this opaqueness makes it harder for SOEs to complete their cross-border deals.

A more nuanced aspect of home institutional effects concerns institutional escape (Cuervo-Cazurra, Luo, Ramamurti, & Ang, 2018; Witt & Lewin, 2007). This argument considers EMFs’ outward FDI as a strategic response to the challenging home institutional environments. Xia, Ma, Lu, and Yiu (2014) find that state ownership can dampen this escape pressure by enhancing local firms’ power and resources. Similarly, China-based FDI flows to international tax havens (a form of institutional escape or arbitrage) is found to be most prevalent for private firms lacking domestic political capital, but least for central SOEs (Deng, Yan, & Sun, 2020). However, studying state-owned R&D entities in India, Choudhury and Khanna (2014) explore SOEs’ motives in seeking resource independence and autonomy from other state actors through globalization. Thus, SOE internationalization may also be related to escaping from firms’ highly dependent relationships with domestic political actors. A recent longitudinal study of a partially privatized Brazilian SOE reveals a dynamic process featuring changing power relationships between the SOE and the home political actors. The process created additional dependencies on the home state, resulting in increasing government control and even de-internationalization (Rodrigues & Dieleman, 2018).

Moving beyond emerging markets, Estrin, Meyer, Nielsen, and Nielsen (2016) conduct a cross-national study on how home institutions affect the internationalization levels of privately owned enterprises and listed SOEs. They find that effective home-country institutional controls enable the convergence of internationalization strategies of the two types of firms. In the context of OECD countries, Mariotti and Marzano (2019) employ the variety of capitalism perspective to compare the degrees of SOE internationalization in liberal, coordinated, and state-influenced market economies. They contend that home-country institutional contingencies can reconcile the theoretical dichotomy between the “government as strategist” and the “liability of stateness” views (c.f. Cuervo-Cazurra & Li, 2021). Additionally, Karolyi and Liao (2017) use the autocracy–democracy classification to examine foreign acquisitions by SOEs, finding that SOEs from autocratic countries are more likely to engage in cross-border acquisitions.

Research from the *host-country institutional perspective* highlights the liability of state ownership (in addition to the standard liability of foreignness). Specifically, the academic discourse emphasizes the lack of legitimacy and the potential distrust/hostility in host countries, especially SOEs from emerging economies (Cuervo-Cazurra, 2018). Meyer, Ding, Li, and Zhang (2014) show how Chinese SOEs addressed their legitimacy issues by adapting their entry modes to host countries’ rule of law and shareholder protection, trading ownership for legitimacy. Grøgaard, Rygh, and Benito (2019) echo this finding, concluding that SOEs took lower ownership shares in acquired firms/assets than private firms, particularly when home countries had lower government quality and less market orientation. Using the US as the host-country context, Li, Xia, and Lin (2017) compare the likelihood and duration of cross-border acquisitions by SOEs and MNEs, finding that SOEs are less likely to complete transactions and, when they do, take longer. Yet, acquisition and alliance experiences in the US market helped mitigate this liability of stateness.

At the *supranational institutional level,* academic inquiries focus on bilateral country relationships and the associated geopolitical issues. Shi, Hoskisson, and Zhang (2016) introduce a broad geopolitical perspective, proposing that the hostility against foreign SOEs is greater when there is a higher degree of religious and political regime dissimilarity, and a lower degree of resource complementarity between home and host counties, with the effects further amplified by host-country nationalist politics. Strong bilateral relationships can have a greater effect on SOEs than private MNEs in mitigating host-country expropriation risk (Duanmu, 2014). Furthermore, Li and colleagues (2018) combine home, host, and bilateral factors to examine the location choice of Chinese state multinationals. While good diplomatic relations induce Chinese firms to invest, this diplomacy effect is particularly strong when the firms are SOEs controlled by the central government investing in a country with a low level of institutional impartiality. Conversely, Chinese SOEs are more likely than their private peers to reduce their investment in countries that have strong political proximity to the US, indicating a clear form of SOE-led economic diplomacy (Duanmu & Urdinez, 2018).

At the intra-organizational level, we did not find any studies that explicitly addressed the interplay between SOE headquarters and subsidiaries in formulating their internationalization strategy, potentially because SOE subsidiaries are less transparent than their private counterparts. Also absent are studies on the roles of SOE TMTs and middle managers in shaping internationalization strategy and outcomes.

#### Managing MNE–host government relationships

The IB field has long studied MNE and host-country government bargaining through the lens of the obsolescing bargaining model (OBM); it theorizes that MNEs entering markets initially possess strong bargaining power that declines over time, because fixed asset investment has become largely sunk, resulting in further misappropriation by host governments (Ramamurti, 2001; Vernon, 1971). Building on Vernon’s earlier framework (1971), subsequent developments and extensions in the IB field include Ramamurti (2001), who incorporated supranational institutions, and Teegen, Doh, and Vachani (2004), who incorporated nongovernmental organizations (NGOs). As such, there has emerged a broader “iterative” model of political bargains negotiated between MNEs and governments over a wide variety of government policies at the industry level (Eden, Lenway, & Schuler, 2005), capturing a more dynamic, multiparty bargaining framework (Müllner & Puck, 2018), and reflecting instances in which a unified one-party bargaining framework is employed, such as in the case of the Chinese government representing the collective interests of Chinese natural resource firms to host-country governments (Li, Newenham-Kahindi, Shapiro, & Chen, 2013).

With respect to *host-country political institutions*, the literature transcends the conception of a monolithic host government by explicating the functioning of specific political institutions (Medina, Bucheli, & Kim, 2019). Henisz and Zelner (2005) develop a neoinstitutional model of the policy-making process, wherein foreign investors engage with host-country interest groups to construct/frame the legitimacy of emergent institutions and impact their trajectories. The model stresses interest group politics and effective checks and balances in host political institutions, highlighting the relevance of firm-specific capabilities and organizational linkages in shaping the bargaining outcomes. The “political bargaining model” developed by Eden et al. (2005) echoes this, recognizing that MNEs employ a wider range of strategies in their evolving relationships with host governments than the conventional OBM suggests.

A stream of empirical studies examining the coopetition between MNEs and host-country governments have also emerged (Luo, 2004; Luo, Zhang, & Bu, 2019). Luo (2001) proposes a cooperative view of the MNE–host government relationships composed of four building blocks: resource complementarity, political accommodation, organizational credibility, and personal relationships. Using survey data from China-based MNEs, he confirms the importance of these elements in building cooperative relationships with regional and national governments, which in turn lead to better organizational performance. Subsequent longitudinal qualitative studies (Frynas et al., 2006; Sun et al., 2010) examine how MNEs develop constructive relationships with host governments. Frynas et al. (2006) highlight the case for developing firm-specific political resources to secure first-mover advantages in host-country markets, as the formation of industry structures and regulatory policies is highly sensitive to skillful political engagement. Sun et al. (2010) use the context of joint ventures with host-country SOEs to assess the effectiveness of foreign partners embedding themselves in domestic political networks in managing their ongoing resource exchanges with political stakeholders.^4^

Some scholarship has sought to integrate *home-country institutions, bilateral government relationships, and supranational institutions.* Ramamurti (2001) proposes a two-tier bargaining model in which home and host governments first bargain bilaterally or through multilateral institutions like World Bank and WTO. He argues that the tier-1 bilateral bargaining can have profound bearings on the tier-2 traditional bargaining outcomes. However, subsequent empirical tests of the model’s major propositions are lacking.^5^ Li and colleagues (2013) revisit this two-tier bargaining model through a qualitative study of Chinese MNEs’ investment in Africa’s natural resource industry. The study suggests a modified two-tier model to underline the central role of the Chinese government in orchestrating the MNE–host-government bargaining process. Overall, similar to table 1 in Müllner and Puck (2018: 16), our review suggests few quantitative empirical works that have systematically tested these revised bargaining models. While a comprehensive evaluation regarding the paucity of the empirical tests is beyond the scope of this review paper, we speculate about some contributing factors. First, it can be challenging to obtain requisite disaggregate data in this multiparty, dynamic bargaining setup. Second, scholars have increasingly adopted a cooperative or embeddedness view of the evolving MNE–host government relations to guide their empirical research design, without necessarily invoking this strand of bargaining-based literature.

Moving to the intra-organizational level, we find little research on the interplay between MNE headquarters–subsidiary relationships and an MNE’s engagement with host-country governments, although some early works have distinguished parent- and subsidiary-level political strategies and explored their interactions (Blumentritt & Nigh, 2002; Hillman & Wan, 2005). One exception is Barron, Pereda, and Stacey (2017), who studied Toyota and Hyundai’s government-affair subsidiaries’ lobbying activities in the EU. They found that organizational design affected the lobbying efficacy of government affairs subsidiaries through the development of managerial social capital in the host region, with Toyota’s decentralized and coordinated government-affairs subsidiaries outperforming Hyundai’s centralized and loosely coordinated subsidiaries.

We saw no explicit attempts to incorporate individual-level factors such as managerial cognition into the bargaining models. The closest research comes from Lubinski and Wadhwani (2020), who examine how Siemens and Bayer capitalized on the growing anti-colonial/nationalist sentiments among Indian political actors to obtain more favorable treatment from the host government. In effect, while studies on TMT political ideology have grown (e.g., Chin, Hambrick, & Treviño, 2013), we do not know much about its effect on MNE–host government relations.

#### Political risk/hazard management

Political risk is a dominant topic in the multinational CPA literature, emphasizing MNEs’ management of host-country political hazards. This has to do with the conventional OBM that advises MNEs to caution against the opportunism of host-country political actors. Most studies focus on host-country political institutions, with some recent works examining the influences of home and supranational institutions on political risk management.

*The host-country institutional perspective* features prominently in this stream of research because MNEs must manage the hazards rooted in host sociopolitical institutions. Building upon the organizational learning perspective, Delios and Henisz (2003) find that firms that are more experienced with a politically hazardous environment are less sensitive to the potential negative impacts of host-country political risks. Furthermore, in some politically salient regulated industries, MNEs initially may prefer to enter countries with a high policy risk, defined by the host state’s discretionary policymaking capacities, since the host is able to offer favorable entry conditions (García-Canal & Guillén, 2008). However, the accumulation of foreign experience of post-entry policy reversals and expropriation can lead the firms to become more averse to politically risky countries over time.

A notable feature of this literature is the development of the “political capabilities” concept to describe firm-specific capabilities to assess/manage political risk and shape policy environments to its own benefit (Holburn & Zelner, 2010). Recent research explores the transferability of risk-management capabilities across borders. For instance, Oh and Oetzel (2017) study if and how MNEs can leverage their experience with political risk (violent conflicts) across borders. They find that only country-specific experiential knowledge about how the host-government manages such conflict risks can help focal MNEs survive a violent environment, whereas the generic experience of dealing with similar conflicts in other countries does not help much. This notion of experiential knowledge and the associated learning mechanisms offer crucial theoretical insights into political risk management and political capability development/deployment, but these angles are surprisingly rare in other parts of the multinational CPA literature reviewed.

As important as the insights from organization-level capabilities are the managerial perceptions of political risk and environment because they shape political risk assessment and strategy formulation. For example, Giambona, Graham, and Harvey (2017) stress the importance of measuring executives’ subjective perceptions of political risk, and link them to corporate cross-border decision-making. Maitland and Sammartino (2015) employ a behavioral perspective in their case study of an Australian firm’s high-stakes strategic decision in a politically volatile African country, explicating the cognitive processes in which executives develop their strategic maps and suggesting a promising avenue for study of the microfoundations of organizational/managerial political capabilities.

Turning to *home-country political institutions,* Holburn and Zelner (2010) suggest that MNEs’ political capabilities of navigating host-country political environments have to do with their home-country institutional arrangements. That is, firms from countries featuring weak institutional constraints on policymakers or strong political rent seeking may seek out riskier host countries for their investments to leverage their political capabilities. Stevens and Newenham-Kahindi (2017) argue that host-country stakeholder perceptions of MNEs are closely related to their home-countries’ legitimacy, resulting in cross-country legitimacy spillovers. In other words, firms must engage with home institutions appropriately, as that can impact their acceptance by host-country government and society, and reduce political risk. Similarly, Han, Liu, Gao, and Ghauri (2018) find that Chinese MNEs regarded their home-country institutions as a major political risk in the EU but not in African countries. These findings suggest a strong connection between host-country political risk and the legitimacy of home-country institutions. Nevertheless, more research is needed to improve our understanding of home-country institutions; in particular, we still know little about how normative and cognitive institutions impact a firm’s political risk in host markets.

At the *supranational institutional level,* home-host-government relations can be an important factor shaping an MNE’s evaluation of political risk. De Villa et al. (2019) find MNEs are likely to elect a non-engaged approach to CPA when they face high political risk, especially as the “distance” between their home and host government relations increases. Moreover, Hasija, Liou, and Ellstrand (2020) show that a high degree of political affinity – defined as a strong similarity in national interests – reduces legitimacy concerns facing MNEs during the post-acquisition integration. This raises the importance of formal political affinities (e.g., international trade rules) and informal ones (e.g., historical ties between countries) and demonstrates how they constrain or enable MNEs to manage policy risks. Jandhyala and Weiner (2014) contend that international investment agreements reduce political risk by limiting the ability of host governments to make discriminatory policy changes. Despite these achievements, the extant literature lacks supranational-level studies evaluating the exogenous political challenges such as Brexit and the US-China trade war. Future research can integrate international politics to study how MNEs address these geopolitical risks (Phan, 2019).

Finally, host-country political risk can be addressed by the configurational design of global firms. Feinberg and Gupta (2009) find that foreign subsidiaries increase the extent of their intra-firm trade in riskier host countries. Yet, we are unaware of research that explicitly examines the interplay between headquarters and subsidiaries with reference to political risk management.

#### Corporate political ties

Research on corporate political ties highlights the antecedents and contingent value of political ties in dealing with institutional complexity abroad (Cui, Hu, Li, & Meyer, 2018; Rajwani & Liedong, 2015; Sun, Mellahi, & Wright, 2012). Such contingency lies in a variety of home- and host-country sociopolitical institutions. We distinguish ties to host-country and home-country political actors in our analysis.

First, *the host-country institutional perspective* plays a dominant part in this research theme. Nurturing connections to host political actors is a crucial strategy to manage MNE–host-government relations. Several recent studies have used cross-sectional data in developing countries to examine the determinants of political tie formation/intensity (Liedong & Frynas, 2018; White, Boddewyn, Rajwani, & Hemphill, 2018a, White, Fainshmidt, & Rajwani, 2018b). They find that MNE political networking is a strategic response to institutional voids in host markets, and that a low level of political and administrative distance between home and host countries will reduce this strategic necessity.

Regarding the consequences of political ties, Sojli and Tham (2017) find that MNEs’ foreign political ties facilitate their entry into host markets; however, other research suggests these effects are contingent. Hitt et al. (2006) examine a sample of US-based law firms and find that the relational capital derived from a firm’s foreign government clients benefited firm internationalization but harmed financial performance. Siegel (2007) suggests that the value of political connections hinges upon the stability of the host political regime: a sudden loss of power by political elites to which a foreign firm is connected can damage the international alliances formed with the foreign firm.

To deepen the understanding of how such contingencies are regulated, we need more longitudinal research to track MNEs’ dynamic interactions with multiple host-country political stakeholders (Bucheli & Salvaj, 2018), including research on political ties from mid-level managers and subnational-/grassroots-level political actors. We also have a limited understanding of how distinct types of political ties evolve over time between MNE headquarters/subsidiaries and specific host-country institutional actors (e.g., trade associations and local councilors). In this regard, Hoang (2018) offers a rich account of how different types of market players structured their relationships with host political actors based on their varied proximity to state officials in Vietnam. This longitudinal qualitative work reveals that local and distinct types of foreign firms vary in their willingness and ability to develop strong political connections with host-state officials; this variance in turn shapes the formation of different MNE strategies in the same institutional environment.

Second, MNEs can develop various network ties to *home-country political institutions*, but research on their impacts on firm internationalization is still emerging. Recent studies have assessed the extent to which home political connections can facilitate firm internationalization (e.g., Fernández-Méndez, García-Canal, & Guillén, 2018), and have explored the interactive effects of political ties and state ownership in shaping EMFs’ internationalization activities (e.g., Liang, Ren, & Sun, 2015). However, we still lack detailed understanding about the processes and outcomes of home-based political connections transferring across national borders. To what extent do these political connections become liabilities when EMFs invest in other economies? If the focal firms are subject to international politics and volatile bilateral home-host relationships, how could MNEs manage such political liabilities of foreignness? Do home-based managerial connections in EMFs experience legitimacy deficits, as state-controlled multinationals do overseas? In brief, we need more research into these diverse types of political linkages with various home institutions.

Third, at the *supranational institutional level,* how MNE political ties interact with supranational institutions to protect resources or offset the risks of investing abroad is an important research question. For example, Albino-Pimentel, Dussauge, and Shaver (2018) find that supranational safeguards in the form of bilateral investment treaties promote foreign investment in the host country. However, companies with superior nonmarket capabilities such as political connections are insensitive to such safeguards, in that they may invest in countries without the bilateral investment treaties. By studying how MNEs build political relationships and interact with supranational institutions, we can generate a more complete picture of MNE political tie utilization and its impacts on firm outcomes.

At the intra-organizational level, once again, there were no studies in our dataset that explicitly distinguish or compare the respective political ties embedded in headquarters and in subsidiaries. While it is normally assumed that ties to host-country political actors are developed and maintained at the subsidiary level, MNE headquarters may also hold political connections to the host state. It would be useful to explore the configuration of MNE political ties and compare the two types of ties in relation to their impacts on firm outcomes.

#### Overall assessment

Multiple institutional embeddedness is a defining feature of MNEs competing across borders (Meyer et al., 2011). Our review underscores the complexity of the nonmarket institutions that MNEs need to tackle across host-country, home-country, and supranational dimensions. While bargaining and engaging with host political institutions and actors remains a central research concern, the burgeoning studies of SOE internationalization strategy over the last decade indicate the relevance of home-country political institutions in shaping EMFs’ strategic actions. Bilateral home-host relationships also emerge as a crucial institutional contingency in current research. Increasingly, research aware of this institutional duality/multiplicity concern (Kostova et al., 2008) has incorporated more than one of the three dimensions in its independent and/or moderating variables to examine the determinants and consequences of multinational CPA. Nevertheless, intra-organizational-level research on headquarter–subsidiary interactions or TMT characteristics is still in its infancy. This leads to a black box of political strategy formulation and implementation within MNEs.

### Multinational SCSR Research

Table 4 lists the top 10, and Online Appendix Table G shows the complete list, of the most cited SCSR-related articles in our dataset. SCSR has been a growing stream within multinational NMS research since 2010, as MNEs increasingly engage with different communities and societal stakeholders as part of their internationalization activities, and those stakeholders exert increasing pressure on MNEs to improve their social and environmental practices. Substreams of SCSR scholarship are not as clearly defined as those of CPA research, possibly because the core research questions are not yet firmly established. Moreover, much of this research considers impacts of home- and host-country institutions simultaneously, as MNEs seek to overcome home-country liabilities via host-country practices, sometimes through the adoption of a supranational institutional arrangement. Further, a number of the most highly cited articles in this category are “perspective” (Kolk & Pinkse, 2012; Teegen et al., 2004) or theory development contributions (Gardberg & Fombrun, 2006), and these contributions provide broad, integrative surveys of a topic, considering multiple sets of institutions, actors, and organizations.

**Table 4:** Top 10 most highly cited articles on international SCSR ranked by Web of Science citations

	Authors (year)	Article	Topic(s)	Type	Theory(ies)	Total cites	Cites/year
1	Maignan and Ralston (2002)	Corporate social responsibility in Europe and the US: insights from businesses’ self-presentations	Comparative CSR	Empirical (quantitative)	Strategic CSR literature	613	32.3
2	Christmann and Taylor (2001)	Globalization and the environment: determinants of firm self-regulation in China	Environmental issues	Empirical (quantitative)	Environmental regulation literature	532	26.6
3	King, Lenox, and Terlaak (2005)	The strategic use of decentralized institutions: exploring certification with the ISO 14001 management standard	Environmental issues; ISO certification	Empirical (quantitative)	Institutional theory and TCE	440	27.5
4	Christmann (2004)	Multinational companies and the natural environment: determinants of global environmental policy	MNEs’ global environmental policy	Empirical (quantitative)	Stakeholder theory	380	22.4
5	Jackson and Apostolakou (2010)	Corporate social responsibility in Western Europe: An institutional mirror or substitute?	Determinants of CSR practices	Empirical (quantitative)	Neo-institutional theory and comparative institutional analysis	353	32.1
6	Gardberg and Fombrun (2006)	Corporate citizenship: creating intangible assets across institutional environments	Corporate citizenship (SCSR)	Conceptual	Institutional theory; Theory of strategic balance	351	23.4
7	Husted and Allen (2006)	Corporate social responsibility in the multinational enterprise: strategic and institutional approaches	Organizational CSR	Empirical (quantitative)	Institutional theory	347	23.1
8	Christmann & Taylor (2006)	Firm self-regulation through international certifiable standards: determinants of symbolic versus substantive implementation	ISO certification	Empirical (quantitative)	TCE	337	22.5
9	Teegen et al. (2004)	The importance of nongovernmental organizations (NGOs) in global governance and value creation: an international business research agenda	NGO	Conceptual (perspective paper)	NA	318	18.7
10	Strike, Gao, and Bansal (2006)	Being good while being bad: social responsibility and the international diversification of US firms	CSR/CSiR	Empirical (quantitative)	RBV	305	20.3

The citation data were collected from the Web of Sciences in mid-February 2021.

*CSR *Corporate social responsibility, *CSiR* corporate social irresponsibility, *FDI* foreign direct investment, *RBV* resource-based view, *RDT* resource dependency theory, *SCSR* strategic corporate social responsibility, *TCE* transaction cost economics.

Given this, we group several related topics in defining three broad research streams: sustainability, standards, and CSR reporting; NGOs, supply chains, and human rights; and corporate citizenship and philanthropy. These three areas reflect the diversity of scholarship in this arena, with the first area constituting the largest proportion of the most highly cited work, while the latter two reflect more emergent areas that require further attention. A number of authors, notably Ans Kolk, have contributed to several of these streams (Kolk & Pinkse, 2007; Kolk, 2010, 2016; Pinkse & Kolk, 2012).

#### Sustainability, standards, and CSR reporting

Of the top ten most-cited articles in our sample, four addressed some aspect of environmental sustainability practices or standards (e.g., King et al., 2005; Christimann, 2004). Those areas constitute a smaller portion of our overall sample but appear to be especially influential. A consistent theme in this area is the use of sustainability practices, accession to sustainability standards, and CSR reporting as a means to overcome home- or host-country liabilities. Kolk (2010), for example, documents the trajectories of sustainability reporting, finding that certain sectors (oil and gas, chemicals) are more likely to publish sustainability reports, and that European firms generally lead those from other parts of the world in the propensity to issue sustainability reports.

One set of articles emphasizes the process by which firms overcome home-country liabilities and establish legitimacy by demonstrating CSR and sustainability commitments (e.g., Marano et al., 2017). This broad area integrates with IB perspectives on institutional adaptation, signaling, and both home- and host-country influences. One variant focuses on efforts by emerging market firms to overcome discrimination associated with their emerging market status by showing how CSR and sustainability signaling serve as a legitimation strategy for MNEs when entering foreign markets (Marano & Kostova, 2016; Marano et al., 2017; Rathert, 2016). Much of this research combines attention to home-, host-, and supranational institutional pressures. For example, Marano et al. (2017: 386) find that emerging market MNEs from less institutionally developed home countries use CSR reporting to convey “alignment with global meta-norms and expectations.” Drawing on neo-institutional theory, they suggest that MNEs’ CSR decoupling is shaped by their dual embeddedness in their *home and host countries* and the global *supranational* institutional environment, a finding echoed in a follow-up study (Tashman et al., 2019). This raises several questions for future research: does dual embeddedness facilitate institutional arbitrage in a form of stronger adaption? How does legitimacy diffuse from home to host and between and among host markets?

Another set of related studies explores the degree to which MNEs are influenced by their global home- and host-country footprint, and whether they standardize their environmental practices globally or adjust them to host-country pressures. These studies show how MNEs themselves can serve as channels through which global social or environmental norms are transferred to developing and emerging economies through FDI. Christmann and Taylor (2001) use survey data from firms in China to show how multinational ownership, multinational customers, and exports to developed countries increase self-regulation of environmental performance. Similarly, Christmann (2004) demonstrates how a range of perceived and actual stakeholder pressures influence global standardization of environmental practices (ISO-14000) among MNEs, finding that perceived international government cooperation in environmental issues and perceived industry pressures both contribute to environmental self-regulation. Interestingly, customer pressures do not directly affect environmental practices, but influence the propensity of MNEs to standardize their *communications* around environmental practices. This suggests that different stakeholder groups elicit different MNE sustainability practices, and that, in some contexts, there is a gap between substantive and symbolic sustainability (Christmann & Tayler, 2006).

Kolk and Pinkse (2008) propose that MNEs can develop firm-specific advantages related to climate change practices that can contribute to profitability, growth, and resilience. They argue that differentiation associated with climate leadership can strengthen the MNE’s position in markets sensitive to climate risk. On the other hand, Husted and Allen (2006) apply the strategic logic of Bartlett and Ghoshal’s typology to explore how MNEs organize their international environmental practices. They find that MNEs generally place substantial importance on global *supranational* environmental issues, but that multidomestic and transnational MNEs place greater importance on country-specific environmental CSR. Child and Tsai (2005) examine similar dynamics and found that MNEs pursue environmentally responsible policies in emerging economies, even when the institutional pressures in those jurisdictions do not compel them to do so. This tendency emerges from the dynamic interplay among MNEs and home and host governments to maintain high environmental performance as well as among the MNEs themselves through networks that represent home-country interests, like the American Chamber of Commerce. Given this, we believe research should better explain how MNEs' global strategy and footprint manifest specifically in their CSR and sustainability practices, including how they signal those practices through reporting or standards adoption.

A related research stream highlights the importance of acceding to *supranational institutions*, such as international private regulatory regimes, to overcome liabilities in host countries and bolster legitimacy (Gifford & Kestler, 2008; Tan, 2009). Pinkse and Kolk (2012) examine how climate change impacts MNEs, proposing that variance in climate-related institutions in home, host, and supranational contexts influences the degree and nature of MNE responses. They suggest that MNEs “face a complex balancing act, concerning embeddedness (or lack thereof) in home, host and supranational contexts, as there are multiple institutional factors that play a role in developing a competitive advantage” (Pinkse & Kolk, 2012: 338). King et al. (2005) explain that firms are more likely to pursue international ISO 14000 environmental standards when they face buyers that are more physically distant and located in foreign markets, because those buyers are less able to acquire information about the supplier or have greater reason to fear opportunistic behavior on their part. In their account, certification serves as a proxy for credibility and legitimacy for suppliers.

Combining attention to home- and host-country pressures, Husted, Monteil, and Christmann (2016: 382) study mimetic effects in sustainability certification among multinationals and local firms, finding that “MNE subsidiaries imitate national certifications by geographically proximate firms to overcome a liability of foreignness, while domestic firms imitate global certifications by proximate firms to overcome the disadvantages of localness.” However, we still know little about how this legitimacy creation unfolds and differs with the particular supranational institution that an MNE adopts. Specifically, how does adoption of these private regulatory institutional standards generate legitimacy in host markets? Further, how do supranational-level legitimacy spillovers change over time and how are they sustained?

Not all of this research presumes that these home- and host-country pressures result in upward harmonization of CSR and sustainability practices. Strike et al. (2006) suggest that firms can be simultaneously socially responsible and irresponsible, finding that international diversification initially results in greater CSR, but that the added complexity and coordinating challenges of diversification lead to socially irresponsible behavior. Surroca, Tribo, and Zahra (2013) explore how stakeholder pressure in an MNE's home country leads to the transfer of socially irresponsible practices from its headquarters to its overseas subsidiaries. This transfer is more pronounced when it is through interlocked, minority-owned subsidiaries, when the institutional environment of the MNE's home country enforces compliance, and when the degree of institutional enforcement for noncompliance in the subsidiary's host country is low. Another contribution suggests that multinational firms face higher CSR expectations than national firms: Zyglidopoulos (2002) proposes that international reputation side effects and foreign stakeholder salience result in greater pressure on MNEs to demonstrate environmental responsibility than on national firms. There is evidence from the social movement literature that activists “target” more visible firms, those in high-polluting industries, but also those that have already made a claim to high levels of social responsibility and sustainability. A potential research question is, how might international diversification or other reflections of internationalization interact with these other factors in drawing critics and activists to a given MNE?

The preponderance of SCSR research in this area does not observe nor measure the actual nonmarket strategies of firms; rather, the strategic intent of these actions is inferred or imputed. Actions such as reporting CSR are presumed to be part of a deliberate strategy to overcome information asymmetries and gain legitimacy, but scholars have been mostly unsuccessful in determining the agency of intent. As such, future research should seek to directly measure the strategic agency of firms as they leverage their CSR activities for strategic benefit. Clearly, a focus on TMT decision-making, HQ–subsidiary directives (Meyer et al., 2020), and other opportunities to directly capture strategic decision-making around SCSR would be a helpful complement to the literature.

#### NGOs, supply chains, and human rights

There is a small but growing body of research exploring the role of global and local NGOs in international business (Kourula, 2010; Teegen et al., 2004). This research stream tends to incorporate *home, host, and supranational* pressures and influences. Teegen et al. (2004) were among the earliest IB scholars to explicitly examine the role of NGOs in IB and global governance. They argue that the emergence of NGOs, especially those with global reach, represents an important complement to traditional IB research that historically focused on firm–firm and firm–government interactions, but not interactions with NGOs. They suggest that the inclusion of NGOs in IB institutional fields is overdue, and that NGOs can exert stakeholder pressures on firms and help create value through their interactions with MNEs. Vachani, Doh, and Teegen (2009) focus on the potential of NGOs to affect the transaction costs of MNEs in several areas: institutional development, institutional distance, and institutional dynamism, all moderated by the growth in civil societies. While NGOs are the subject of research in political science and sociology, IB scholars have yet to take up the call from Teegen et al. (2004) to focus on them as the principal subject or actor in IB. Yet, many NGOs demonstrate analogous qualities to MNEs in that they manage across borders, cultures, and institutions; feature international governance and operational challenges; and formulate strategic goals and establish performance measures in their international operations. Further, both private MNEs and not-for-profit NGOs are led by individuals who themselves influence the global direction of these organizations.

Although sustainability in supply chains has gained considerable practitioner and policy interest, as home-country institutions have pressured firms to better demonstrate the social and environmental practices of their suppliers, just one of the most-cited articles address issues related to CSR and sustainability in global supply chains. Kim and Davis (2016) use the natural experiment of Section 1502 of the Dodd–Frank Act of 2010, which required MNEs headquartered in the US to report efforts to certify the origin of the minerals they used in their production, to examine the factors that help or impede companies’ efforts to provide this information. They report that 80% were unable to determine that the host-country origin of their suppliers was conflict-free. As firms became more internationally diversified with more dispersed supply chains or outsourced their operations, the ability to trace and report this information became even more elusive. Another article makes the argument that the universe of suppliers that MNEs are now responsible for certifying includes all relevant contractors and producers, expanding the scope of responsibility and placing greater pressures on the MNE to provide assurances to governments and other stakeholders (Schrempf-Stirling & Palazzo, 2016). This stream not only underscores the growing pressures on firms to certify the social and environmental practices of their supply chains and the potential benefits of doing so, but also highlights the practical challenges associated with reaching back into the supply chain to gain this information, and to influence suppliers to comply with focal firm practices.

Another related and relatively underrepresented topic among the most-cited articles is the roles and responsibilities of MNEs with regard to human rights, workforce practices, and labor relations. Again, here it is primarily home-country pressures that push MNEs to improve host-country practices. Since human rights emerged as a critical concern of MNEs, NGOs, and governments, research on the relationship between business and human rights has attracted substantial interest among legal and management scholars, but has not fully penetrated IB. Indeed, “the discussion on business responsibilities for human rights is thriving – although, surprisingly, predominantly outside of the International Business (IB) field” (Wettstein, Giuliani, Santangelo, & Stahl, 2019: 54). Given the continued concern about issues such as working conditions in factories around the world and the incorporation of human rights and labor practices in major trade agreements, this would seem like a natural realm for IB scholarship.

In short, we see an underrepresentation of research related to NGOs, sustainable supply chains, and human rights in SCSR research, and we believe a number of important research questions arise. First, how should MNEs decide which NGO(s) to partner with? Given the absence of independent NGOs and the prevalence of government-organized NGOs in China, how do partnerships with the latter differ from independent NGOs? How can MNEs use their individual and collective influence to ensure that their suppliers adhere to the focal firms’ labor and human rights standards and what benefits of doing so accrue to them? What new institutions may emerge to reconcile global labor and human rights practices with local ones and how can MNEs shape and influence the emergence of those institutions?

#### Corporate citizenship and philanthropy

Research on “corporate citizenship” practices and international corporate philanthropy suggests that philanthropic donations can help organizations build resilience and strengthen relationships with local stakeholders (Hornstein & Zhao, 2018). Brammer, Pavelin, and Porter (2009) find that corporate charitable giving increases with internationalization, and that MNEs give more in countries that lack political rights and/or civil liberties, speculating that this is both to fill institutional voids and to enhance marketing strategies in those countries.

At the *home institutional level*, corporate citizenship programs that include philanthropic donations can improve stakeholder relationships at home that can then support MNEs abroad. Bhanji and Oxley (2013) suggest that private investments in home-market public goods can also impact firms in host markets, but that donations from corporations face both a liability of privateness in that public and private actors are skeptical of corporate involvement in such initiatives, and a liability of foreignness when those initiatives are part of international strategies. These insights raise the importance of philanthropic governance mechanisms and MNEs’ alignment with different strategic partners.

At the *host-country level,* Gardberg and Fombrun (2006) explain that local institutional environments shape expectations of corporate commitment to citizenship programs and can help firms overcome barriers in different local markets. Zhang and Luo (2013) adopt a social movement perspective on MNEs’ responsiveness to social issues in emerging markets, focusing on how online activists secure firms’ attention to provide philanthropic contributions. Their empirical context is corporate philanthropic action following the 2008 earthquake in China’s Sichuan province, which triggered an online campaign pressuring MNEs to donate to the disaster relief effort. Companies responded sooner and at a higher magnitude if they presented themselves as highly committed to CSR, had a high reputation in the host country, were headquartered in countries with an institutional logic of discretionary corporate philanthropy, and were under stronger pressure from the activist campaign. This study integrates several themes of the overall SCSR literature, and is part of a small but interesting set of studies that examine corporate responses to natural disasters, some of which are discussed in the next section, as they integrate both CPA and SCSR elements. Echoing some of the findings of Zhang and Luo (2013), Mithani (2017) finds that strategic philanthropy following a natural disaster was stronger in MNEs versus local firms, and that it helped those firms to establish strong local ties, enhance local acceptance, and mitigate liabilities of foreignness.

There are only five additional articles on international philanthropy in our dataset, all published after 2013. This suggests we know little about the role of corporate philanthropy in nonmarket strategy across different host institutional settings. Given that corporate philanthropy is a crucial form of SCSR, we hope to see more research investigating the role of cross-border philanthropy in shaping MNEs’ competitive position and legitimacy in host economies. For example, how do MNEs coordinate their donations in home- and host-country markets? How do the institutional environments in host countries influence the donation cause? How do firms leverage their nonmarket resources and capabilities to strengthen the impact of donations through volunteer programs and NGO partnerships?

#### Overall assessment

In reviewing the body of NMS literature related to SCSR, we find that research is still nascent with few clear and well-established research streams. Further, many of the contributions are broad surveys, incorporating a wide range of institutional considerations and stakeholders. Perhaps even more than in the CPA literature, this research suggests MNEs find themselves facing pressures, constraints, and in some cases opportunities from multiple institutional levels: home countries, host countries, and supranational institutions, often using the latter to bridge those disparities. Perhaps due to limited availability, many studies rely on somewhat limited sources of data. Finally, as was the case with the CPA literature, we fail to find in-depth studies dedicated to examining the roles of HQ-subsidiary interactions and individual executives in SCSR formulation and implementation.

### Integration of CPA and SCSR

The emerging theme of CPA–SCSR integration accounts for less than 20% of the total articles in our dataset; none of them were published before 2006, the year when *JIBS* first advocated for this integration in its special issue (Rodriguez et al., 2006). Table 5 presents the top 10 and Online Appendix Table I provides the complete list of most-cited articles in this area, and we identify three major topical issues: concurrent engagement with social and political stakeholders, complementarity between CPA and SCSR, and dealing with corruption.

**Table 5:** Top 10 most highly cited articles on CPA & SCSR ranked by Web of Science citations

	Authors (year)	Title		Topic(s)	Type	Theory(ies)	Total cites	Cites/year
1	Doh and Guay (2006)	Corporate social responsibility, public policy, and NGO activism in Europe and the United States: an institutional-stakeholder perspective		NGO and public policy	Empirical (qualitative)	Institutional theory; stakeholder theory	505	33.7
2	Rodriguez et al. (2006)	Three lenses on the multinational enterprise: politics, corruption, and corporate social responsibility		CPA, CSR and Corruption (Special issue introduction)	Conceptual	NA	241	16.1
3	Luo (2006)	Political behavior, social responsibility, and perceived corruption: a structuration perspective		CPA, CSR and Corruption	Empirical (quantitative)	Giddens' theory of structuration	133	8.9
4	Spencer and Gomez (2011)	MNEs and corruption: the impact of national institutions and subsidiary strategy		Bribery/corruption in host countries	Empirical (quantitative)	Institutional theory	99	9.9
5	Cuervo-Cazurra (2008)	The effectiveness of laws against bribery abroad		Bribery in host countries	Empirical (quantitative)	New Institutional Economics	95	7.3
6	Montiel et al. (2012)	Using private management standard certification to reduce information asymmetries in corrupt environments		Corruption; environmental issues	Empirical (quantitative)	New institutional economics	92	10.2
7	Doh et al. (2017)	International business responses to institutional voids		CPA and CSR	Conceptual (special issue editorial)	NA	87	21.8
8	Stevens et al. (2016)	Toward a legitimacy-based view of political risk: The case of Google and Yahoo in China		Political risk	Empirical (qualitative)	Institutional theory	84	16.8
9	Cuervo-Cazurra (2016)	Corruption in international business		Corruption	Conceptual	Agency theory, TCE, RBV, RDT, and Institutional theory	84	16.8
10	Detomasi (2008)	The political roots of corporate social responsibility		Political determinants of strategic CSR	Conceptual	NA	79	6.1

The citation data were collected from the Web of Sciences in mid-February 2021.

*CPA* corporate political activity, *CSR *corporate social responsibility, *RBV* resource-based view, *RDT* resource dependency theory, *TCE* transaction cost economics.

#### Concurrent engagement with social and political stakeholders

Some IB researchers have adopted a holistic approach to understanding MNEs’ engagement with social and political stakeholders in home and host countries, which is an important initial step in exploring MNEs’ strategic responses to a broad nonmarket environment. In the emerging market context, Boddewyn and Doh (2011) address a key “political CSR” agenda that involves the collaboration of MNEs, NGOs, and host-country governments to provide collective goods in emerging economies.

Focusing on *host-country local stakeholders*, Reimann, Ehrgott, Kaufmann, and Carter (2012) draw on stakeholder theory and the legitimacy perspective to examine the roles of both local employees and local governments in pushing MNE subsidiaries to adopt high standards in their working conditions and to engage in developing the local community. Similarly, when examining MNE responses to host-country violent conflicts, Oetzel and Getz (2012) cover a wide range of social and political stakeholders in host countries – employees, consumers, NGOs, governments, and media at the local and international levels and in the international arena – and investigate how those stakeholders pressure MNEs to develop strategic responses.

More recently, scholars have begun exploring the underlying linkages between *home-country institutions* and MNEs’ *host-country* stakeholder strategies. Carney, Dieleman, and Taussig (2016) develop the concept of cross-border institutional capabilities – defined as heuristics, skills, and routines that can help MNEs effectively engage with the range of social and political stakeholders in host-country institutions – and explicate the process in which an Indonesian company initially developed its institutional capabilities in its home country and then transferred them to Vietnam.

Another conceptual development is to adopt a business ecosystem perspective that treats *home- and host-country* sociopolitical stakeholders as interdependent community members. In this respect, Parente, Rong, Geleilate, and Misati (2019) examine how a Chinese SOE sustained its operations in Congo by coevolving with key stakeholders, including home and host governments, state-owned and private enterprises, and local communities, over different stages of the firm’s development. Thus, the construction of a business ecosystem involving various home- and host-country stakeholders proves a crucial nonmarket strategy to navigate a precarious and challenging host-country environment. However, there are still unanswered questions regarding engagement with sociopolitical stakeholders. For instance, what is the return on investment of these sociopolitical engagements? What balance should MNEs adopt in frequency and mode of engagement with these sociopolitical stakeholders?

As for supranational institutional factors, except for global NGOs, we are unaware of any studies that attempt to explicitly involve multilateral organizations in the focal stakeholder groups with which MNEs engage. When it comes to intra-organizational factors, Caussat, Prime, and Wilken (2019) examine the tensions between French bank headquarters and their India subsidiaries with regard to legitimation strategies directed toward local social and political stakeholders. In sum, this stream of literature recognizes the importance of taking into account both social and political stakeholders in home- and host-country environments when studying MNE stakeholder strategy, but it falls short of revealing the underlying mechanisms that regulate the interplay between political players and other social stakeholders.

#### Complementarity between CPA and SCSR

Research on the intersection between CPA and CSR has recognized that many CSR activities of developed-country MNEs can be inherently political (Detomasi, 2008). Examining the interactions among MNEs, governments, and NGOs, Doh and Guay (2006) explore three case studies on global warming, trade in genetically modified organisms, and pricing of anti-viral pharmaceuticals in developing countries, finding that different institutional structures and political legacies in the US and EU significantly affect how governments, NGOs, and the broader polity decide on the types of CSR to implement.

Research has suggested that SCSR activities can help focal firms obtain legitimacy and resources controlled by political actors, thus generating a complementary relationship between SCSR and CPA (Mellahi et al., 2016). When MNEs navigate challenging *host-country institutional environments*, host-government and political actors remain primary stakeholders and control resources that MNEs seek to acquire. Thus, SCSR activities may become one of the strategic agendas involved in the long-term interactions between MNEs and the host state, as advanced by recent research on using CSR to enhance legitimacy with host governments and to manage MNEs’ political relationships. Specifically, Beddewela and Fairbrass (2016) examine how MNEs can develop community CSR initiatives to engage with governmental stakeholders. Dubbed a “manipulation” strategy, these activities reflect the alignment of community initiatives with government agencies’ objectives in exchange for legitimacy and policy support.

On the other hand, MNEs’ CPA can also facilitate the development of overall stakeholder management in the host country. Mbalyohere and Lawton (2018) offer important insights in their longitudinal study of five MNEs in Uganda’s electricity generation sector. CPA could be leveraged to improve local stakeholder engagement and accommodate multilevel stakeholder pressures (a traditional function performed by SCSR) as pro-market reform deepens. The study develops the notion of “stakeholder-oriented political capabilities” to characterize the synergy between CPA and SCSR.

Another form of complementarity concerns SCSR’s insurance function in the event of exogenous political shocks/hazards. Darendeli and Hill (2016) conduct an in-depth multiple case study exploring how MNEs’ development of complementary nonmarket tactics can weather the storm of political shocks in Libya. They find that firms that had cultivated strong ties to Qadhafi’s authoritarian regime, and also invested in social-benefit projects and social ties with families less tied to Qadhafi, generated greater legitimacy once his regime fell than those that had relied on only one set of political ties. Thus, SCSR activities serve as a crucial hedge against the volatile host political environment that is beyond MNEs’ control.

When we broaden the single focus on *host-country institutions* and incorporate influences from *home-country* contingencies, CPA–SCSR interactions can become challenging. Stevens et al. (2016) unraveled tensions between engaging politically with host-country governments and maintaining legitimacy in home-country societal stakeholders. Using case illustrations of Google and Yahoo in China, they find that, when MNEs establish closer ties to more authoritarian governments, those ties may generate negative effects on legitimacy and social image at home or in other countries. Echoing the relevance of institutional duality/multiplicity facing MNEs, this key insight underscores the tension between a firm’s political and social activities at the global level, in that locally desirable corporate political tactics in the host country can be at odds with the standards of or expectations from dominant stakeholders in the home country.

Similar conflicts may emerge when MNEs from state-capitalist economies meet global NGOs at the *supranational level*. Villo, Halme, and Ritvala (2020) use the Arctic oil drilling dispute between environmental NGOs headed by Greenpeace and Gazprom, the Russian energy giant, to highlight the conflicts between domestic political stakeholders and global social stakeholders. They theorize that MNE–NGO–state conflict escalation can have perverse effects, in that MNEs headquartered in state capitalist economies receive strong government support and are found less susceptible than developed countries to NGOs’ environmental demands, but that this protection results in negligence toward the activist-contested environmental risks.

#### Dealing with corruption

By definition, corruption necessarily involves a mixture of political and social actors. Corporate bribery involving public and private agents is ubiquitous in weak institutions and can have political and ethical repercussions. In the IB context, scholars are primarily concerned with the organizational and environmental factors that shape MNEs’ interactions with and nonmarket responses to corrupt environments in the host economy.

*At the host-country institutional level*, Luo (2006) studies how MNEs react to increased corruption in the Chinese business segment. He finds a bifurcation of MNEs in terms of their strategies. While those having a stronger ethical focus tend to buffer the corruption risk by using arm’s length bargaining with the host government, MNEs that focus less on ethics take advantage of the opportunity by relying on social connections (e.g., political networks) to engage with the host government. Using Ukraine’s service sector as the research context, Rodgers, Stokes, Tarba, and Khan (2019) examine how MNEs navigate the challenging institutional environment by outsourcing “corrupt” activities through third parties to bypass the scrutiny from headquarters and home-country stakeholders. Monteil and colleagues (2012) looked at potential links between SCSR activities and host-country corruption. They find that obtaining ISO certification (third-party endorsement of quality/environmental management) served as a credible private signal to facilitate both domestic firms and foreign subsidiaries in dealing with policy-specific corruption in Mexico. In other words, where the government’s regulatory integrity is lacking, firms tend to use private signaling devices to reduce information asymmetries in market transactions.

The extant research has also examined the antecedents of corruption *at the home-country and supranational levels*. The general consensus is that higher home-county governance quality and multilateral anti-corruption treaties help deter MNEs’ corruption activities abroad. For instance, Cuervo-Cazurra (2008) identifies a significant interaction between home-country legal regulation and the OECD Anti-Bribery Convention, such that MNEs are less likely to invest in corrupt countries when home countries have laws against bribery abroad and are signatories of the Convention. Using the UN Oil-for-Food Program in Iraq as their research setting, Jeone and Weiner (2012) also confirm that firms made fewer bribery payments when their home countries implemented the OECD Anti-Bribery Convention.

Combining the host, home, and supranational dimensions, Spencer and Gomez (2011) examine MNE subsidiaries’ pressures to undertake corrupt practices in emerging and developing economies. They find that the institutionalization of corrupt practices in both host- and home-country environments are primary drivers of subsidiary-level corruption pressures. Further, when MNEs did not have local partners, firms from less corrupt countries had fewer pressures to engage in corrupt practices locally.

Echoing Cuervo-Cazurra (2016: 42), further research on corporate corruption can generate more insights into the company-level control mechanisms; in the MNE context, we need more research to examine the headquarters–subsidiary interactions in relation to controlling corrupt activities. This is also related to the need for more microlevel inquiries into the motivations and perceptions of corruption on the part of different subsidiary and headquarters managers. For example, expatriates and local managers may have different perceptions, motives, and capabilities in relation to corruption control.

Finally, current research is largely silent on the consequences of MNE corruption under weak institutions, such as potential sanctions or reputation losses. While some political scientists have already identified positive associations between MNE investment and host-country rent-seeking corruption activities in countries like Vietnam and China (Malesky, Gueorguiev, & Jensen, 2015; Zhu, 2017), little IB research has explored the dark side of MNE bribery activities in a corrupt host-country environment.

#### Overall assessment

The literature integrating CPA and CSR in the international context is extremely limited. Many research questions are left unanswered or unexplored, which we address in the next section.

## DISCUSSION, FUTURE RESEARCH, AND CONCLUSION

As an interdisciplinary field, IB has long drawn upon conceptual and theoretical insights from other fields. Scholars have observed, however, that, while IB has made important contributions to business management scholarship, it has not been as successful at influencing the broader social sciences fields, such as economics, sociology, and political science, resulting in a “trade deficit” between IB and these other areas (Buckley et al., 2017; Delios, 2017). Opportunities to provide substantive contributions to these broader literatures typically involve borrowing a core theoretical concept and reconfiguring it to assume additional qualities and contingencies in the IB context, as was the case in the development of internalization theory, which was initially inspired by transaction cost economics. Accordingly, we believe the multinational NMS is a fruitful domain to push IB research to draw from, connect with, and contribute to other areas of business management and the broader social and behavioral sciences. The multilevel framework that organizes our integrative review of this diverse body of literature serves as an initial step to capture this potential.

In this final section, we identify four broad thrusts for future research that reflect potential contributions of multinational NMS research. The first three areas build on our review of the existing streams in the literature and propose the integration of theoretical perspectives from outside of IB that can be used to strengthen and enhance those streams. The fourth focuses primarily on changing phenomena and emergent topics that should capture scholarly attention. In each case, we have identified the relevant theoretical insights, proposed sample research questions, and offered an illustrative methodological approach. These areas are admittedly selective and reflect our assessment of fruitful directions. Table 6 offers a summary of these four research areas.

**Table 6:** Future research thrusts in multinational nonmarket strategy

	Future research thrust #1: greater attention to microlevel processes and strategies	Future research thrust #2: greater attention to global macro-level processes, institutions, and interactions	Future research thrust #3: the interaction of CPA and SCSR as complements or substitutes	Future research thrust #4: incorporating multi-actor global issues and movements
Theoretical linkages	Microfoundations of Strategy Upper Echelon Theory Other Behavioral Theories of the Firm	Economic and Corporate Diplomacy Policy Transfer Theory	Instrumental Stakeholder Theory Institutional Theory	Social Movement Theory Social Network Theory
Representative research question	How do MNE executives and TMT influence the configuration of nonmarket strategies at the global, regional, and local level?	How might the global network of American Chambers of Commerce facilitate the transfer of CSR practices from one jurisdiction to another?	How do home, host, and global institutional environments shape the ways in which an MNE combines its CPA and SCSR tactics across countries?	How do MNEs respond to activists that target multiple facilities in different world regions?
Representative methodological approaches	Discourse-based methods	Multilevel Methods	Difference-in-Difference Approach and Qualitative Case Studies	Social Network Analysis

### Future Research Thrust #1: Greater Attention to Microlevel Processes and Strategies

Our review of the multinational NMS literature has exposed the paucity of microlevel research. Specifically, few studies incorporate the decision-making processes undertaken by executives/TMTs, or the microfoundations of the strategy processes, which IB scholars have long bemoaned. Foss and Pederson (2019) argue that the causal claims made by scholars observing macrovariables should be further elaborated by the examination of their constituent components, namely individuals and their interactions.

Kano and Verbeke (2019) assess the behavioral foundations of key IB theories, focusing on two core assumptions: bounded rationality and bounded reliability, showing how the applications of these assumptions and the processes they imply can enrich existing IB theoretical perspectives. One of the main areas is institutional theory, a dominant theoretical perspective in multinational NMS and the foundation of our review. They argue that institutional perspectives are too inclusive in incorporating myriad actors and influences at the home- and especially the host-country level, and they presume a form of mechanistic response by the MNE to these institutional forces. They propose that explicit examination of bounded rationality and bounded reliability can better explicate the processes that lead to observed outcomes, and also provide better explanations for deviations and variations, say, in a subsidiary’s adoption of headquarters policies versus locally induced pressures. They contend that “increased attention to individual agency could also shed light on the specific mechanisms through which firms can alter host-country institutions – an arguably underappreciated aspect in institutional theory research” and “explicitly articulating microfoundations could make institutionalization theory more actionable … by providing structured and specific mechanisms to align individual-level behavior, firm-level strategy, and country-level institutional environment” (Kano & Verbeke, 2019: 141).

In this regard, we know precious little about the microfoundations and decision processes by which companies configure their nonmarket strategies within the global firm. IB has long explored headquarters–subsidiary relationships and subsidiary initiatives (Bouquet & Birkinshaw, 2008), but little of the research we reviewed focused on the configuration of nonmarket strategies – e.g., lobbying or formation of political ties, deployment of stakeholder-directed CSR strategies – at the global, regional, and local levels (for notable exceptions, see Barron et al, 2017; Lawton, McGuire, & Rajwani, 2013a, Lawton, Rajwani, & Doh, 2013b), and almost none examined the microprocesses that undergird those choices (for a notable exception, see Maitland & Sammartino, 2015). It is clear that NMS is especially subject to both global pressures and local responsiveness, given differences in laws/regulations across regions, varying social expectations in different cultural contexts, and stakeholder pressures on MNCs across their global footprint. However, managers, executives, and individual political and nongovernmental agents have decision-making authority. The multinational NMS research has made very limited progress in documenting the roles of individuals in the decision processes that precede the largely latent, observed outcomes. As such, research is overdue in exploring how individual executives allocate responsibility for NMS between global and regional headquarters and subsidiaries, and the power and knowledge that flow between these units and their managers. Relatedly, upper echelon theory has stressed that organizational outcomes are shaped by the backgrounds and characteristics of senior executives (Hambrick & Mason, 1984). Specifically, both demographic and task/experiential characteristics of CEOs and TMTs have been shown to influence a range of strategic decisions of firms. As is the case with microfoundations research, IB has not historically directed much attention to TMT research. One study by Nielsen and Nielsen (2011) finds that internationally diverse teams contribute to organizational performance, but it does not explicitly consider the international strategies of the sampled firms. In this regard, microlevel research that examines educational, experiential, and dispositional aspects of TMT and their impacts on NMS is warranted. For example, TMTs and boards that include executives with extensive international experience and multicultural backgrounds may approach nonmarket strategy differently than those without such experience. They are oftentimes boundary spanners (Caussat et al., 2019; Meyer et al., 2020) who work across different countries, cultures, and MNE subunits, and can generate new organizational dynamics (Han, Jennings, Liu, & Jennings, 2019; Rodgers et al., 2019). Similarly, CEOs and TMT members who themselves have prior experience in the political or NGO world and/or strong political convictions might bring a different perspective to NMS practices within the global firm (Marquis & Qiao, 2020).

In sum, microlevel research has been particularly underrepresented in multinational NMS research. It is for this reason that we explicitly included intra-organizational actors and processes and the TMT in our analytical framework, even though the extant literature has largely left them out. As such, we advocate for greater attention to microfoundations and upper echelon research within the context of institutional perspectives to reveal the often-unobserved dialog, debate, and tensions that precede a decision to configure and execute NMS in the global firm. Such work may require alternative methodological approaches that have not been especially prominent in IB research. Some of this microlevel work may require “rich descriptions” to reveal the longitudinal processes in order to identify key decision points and the actors that contribute to them. In their critique of internationalization theory, Trevino and Doh (2020) argue that discourse-based perspectives could complement traditional international process theory by uncovering what Vahlne and Johanson (2017) themselves referred to as the “black box”, the dynamic internal and external processes leading the firm to an internationalization initiative. They suggest that “rather than identifying and documenting internationalization outcomes, discourse analysis allows for the integration of managerial processes through which organizations evolve and are sustained” (Trevino & Doh, 2020: 2).

### Future Research Thrust #2: Greater Attention to Global Macro-Level Processes, Institutions, and Interactions

A counterpart to the prior discussion is the desirability of incorporating broader macrolevel processes and institutions. Our survey reveals limited attention to comparative NMS practices across different institutional settings and even less focus on international governmental institutions and NGOs.

With regard to the first focus area, one stream of IB research that does speak to this connection is empirical analysis in comparative institutionalism. One of our most highly cited articles (Doh & Guay, 2006) uses this perspective to compare CSR in Europe and North America, while Jackson and Apostolakou (2010) examine CSR practices in different Western European countries. Li, Cui, and Lu (2014) conceptualize diversity between SOEs affiliated with central and local levels of government as a factor in their ability to negotiate for home- and host-country institutional legitimacy. Yet, this literature is quite limited and we see additional opportunities to employ this lens to uncover additional and more specific dimensions of multinational NMS. For example, how might the “varieties of capitalism” among Asian and European economies influence the focus and configuration of NMS? How might companies with dual headquarters in different world regions organize and deploy their CPA and CSR in these and other regions?

Regarding the second area of interest, while the SCSR research does integrate the role of some private regulatory regimes, such as ISO 1400 standards, overall there is inadequate attention paid to these institutions. This may be due to IB scholars’ assumption that scholarship focused on these entities more appropriately falls to political science and international relations scholars. Yet, several traditions in multinational NMS, such as MNE–host-government relations, draw directly from the international relations and global political economy traditions. Building on Dau, Moore, and Newburry (2020), we suggest that future research can draw on international relations scholarship to explore; for example, using diffuse reciprocity (the expectation that there will not be an equivalence of obligations or concessions in any one exchange, but, rather, a balance over an ongoing series of exchanges with a group of partners) to examine the role of SCSR between home and host institutions.

In this regard, one helpful stream of multinational NMS literature is scholarship on international economic diplomacy generally, and corporate diplomacy in particular. Companies assess opportunities and threats that emanate from the nonmarket environment across governance levels, sectors, and issue domains (Henisz, 2016) and they assume an active "foreign policy" stance and articulate a political and social agenda in relation to national and global institutions (Saner & Yiu, 2008). A small number of contributions in our database echo this tradition (e.g., Li, Meyer, Zhang, & Ding, 2018), as they delve into the interplay between bilateral home–host relationships and home–host sociopolitical institutions and examine its impacts on MNE strategic actions, but much of the limited literature is applied and atheoretical. As such, we encourage deeper engagement by IB scholars.

In addition, there are theoretical traditions from political science that could be borrowed and integrated into IB. One such tradition is the *policy transfer* literature, which maintains that policy approaches “transfer” via international organizations and bodies, resulting in policy convergence between and among nations (Evans, 2009). This involves knowledge exchange between national governments and international institutions and advocacy by MNEs and NGOs (Dolowitz & Marsh, 2012; Stone, 2012), giving companies opportunities to influence the evolution of economic policies. One recent practical example is the renegotiation of NAFTA that resulted in the US–Mexico–Canada Agreement. Here, companies sought to influence the negotiations through direct advocacy with their home governments, industry associations with home and host governments, and the regional NAFTA/USMCA institutions through formal solicitations of input.

In addition, emergent policies are “transferred” not just among national and international governmental organizations but also through MNEs and other global actors. Several articles in our database partially adopt this perspective. Pinkse and Kolk (2012) touch on institutional conformity and mimicry among MNEs as they adopted more climate-friendly policies, while Child and Tsai (2006) show how MNEs carry their home-country’s environmentally responsible policies to emerging economies. Other important areas for study might include exploring how the global system of national Chambers of Commerce facilitates the transfer of CSR practices in one jurisdiction (the home market) to others (in the host markets) through the network.

Methodologically speaking, given the importance of understanding nonmarket strategy as it interacts with global, national, and subnational governmental and NGOs, it is surprising how little research has employed multilevel theorizing and method to capture the influences at these various levels. Although Husted et al. (2016) incorporate local and national variables in their examination of sustainability certification by MNEs and domestic firms in Mexico, they do not use hierarchical methods *per se*. Interestingly, research on culture (Autio, Pathak, & Wennberg, 2013) and institutional distance (Hernández & Nieto, 2015) has already embraced multilevel methods. As such, we believe that the application of multilevel methods would be an appropriate approach to examine the influences of the multiple institutional levels captured in our analytical framework, as they involve the dynamic exchange of knowledge and pressures among supranational, regional, national, and local institutions.

### Future Research Thrust #3: Greater Attention to the Complementarity and Tension Between Multinational CPA and SCSR

Given the complexity and connectivity of the nonmarket environment, scholars have long called for examination of the alignment and integration of CPA and SCSR (Baron, 2001; den Hond, Rehbein, De Bakker, & Lankveld, 2014; Lawton, Doh, & Rajwani, 2014; Mellahi et al., 2016). Lawton et al. (2013a, 2013b) argue that the growing breadth of CPA constitutes a mechanism to achieve policy access and to manage a broader set of stakeholders and the range of their external demands of the firm.

Our review indicates that scholars are making increasing efforts in this regard by exploring the ways in which CPA and SCSR interact in a complex global institutional setting. However, we believe more research is needed to explore the complementarity/substitutability between, and the combinative effects of CPA and SCSR across, different home- and host-country contexts. As one example, research can use longitudinal design to study how MNEs adjust their CPA–SCSR combinations given evolving pressures and conditions in home, host, and global contexts.

To this end, we propose a synthesis of stakeholder and institutional theories to serve as a conceptual foundation for unraveling the complex and dynamic interplay between the two strategies. While stakeholder theory in the current context emphasizes MNEs’ identification of stakeholder salience based on the power, legitimacy, and urgency of social versus political stakeholders (Mitchell, Agle, & Wood, 1997), the institutional multiplicity perspective offers a concrete avenue for MNEs to understand various and often conflicting demands/pressures of these stakeholders, which are in turn embedded in a variety of home, host, and supranational institutions.

When powerful political stakeholders control enormous resources in host emerging economies, CSR projects undertaken by MNEs can serve as an inherent political tactic to develop cooperative relationships with the host government, especially in places like China where many NGOs are government-affiliated. While the literature establishes this general insight, we do not know if the “political” CSR activities will be less intensive when political stakeholder power declines. Further research in this aspect can verify the robustness of this theoretical insight and identify its potential boundary conditions. Further, given that MNEs may also use conventional CPA to engage with host political actors, what are the conditions under which an MNE will choose to undertake SCSR, CPA, or both?

Another form of complementarity concerns the insurance role of CSR initiatives in case a focal MNE faces serious political hazards in the host country (Darendeli & Hill, 2016). If host-country CSR initiatives can be treated as an insurance policy acquired to manage political risk, future research needs to evaluate the limit of this insurance effect. How comprehensively and for how long can social capital generated through SCSR activities protect MNEs against expropriation from host-government opportunism? We suspect that the effectiveness is contingent upon a range of institutional and organizational factors that await future investigations.

Concerning the potential tension or internal inconsistency between the two strategies, the global scope of MNEs can result in more tensions and contradictions than in domestic companies. For instance, if multinationals lobby for immigration restrictions in their home countries while donating to social justice causes in other countries, will this inconsistency threaten their reputation in global markets? Our review also suggests that host-country CPA can be misaligned with home-country and global ethical perceptions (Stevens et al., 2016). In view of these potential tensions, future research can employ the institutional multiplicity/complexity perspective to further examine how certain CPA may conflict with other CSR initiatives across jurisdictions and identify the consequences of the misalignment.

With regard to research methodology, we believe a combination of quantitative and qualitative approaches can foster the development of this emerging area. On the quantitative front, research should consider the endogeneity of CPA and SCSR variables. Leveraging (quasi)natural experiments via the difference-in-difference estimation technique is one way to identify causality involved in the interactions between CPA and SCSR. Meanwhile, qualitative case studies are crucial to reveal the operating mechanisms through which CPA complements or substitutes for SCSR activities in varying institutional contexts. Since the topic is relatively new and process-based, a thick description of institutions (Aguilera & Grøgaard, 2019) via qualitative data enables promising theory development (Eisenhardt, 1989).

### Future Research Thrust #4: Incorporating Multi-Actor Global Issues and Movements

There is ample room to broaden the universe of issues, actors, and problems examined by this important research stream. For example, scholars in economics, political science, and sociology have recently come together to explore the multi-actor interactions among social movements and stakeholders – both governmental and nongovernmental – and corporations (De Bakker, den Hond, King, & Weber, 2013; Soule, 2009, 2012; Yaziji & Doh, 2013). Global social movements have exerted pressure on MNEs to improve working conditions, environmental preservation of key resources such as forests, and gender equity. Yet, these topics are little explored in IB. A number of MNEs have been targeted by the same NGO activists, such as when British Petroleum was concurrently developing pipeline projects in Alaska, the Caspian Sea, and Indonesia, and faced opposition from both local civil society groups and globally coordinated ones. This example raises several questions that have important theoretical implications: how do companies coordinate responses at the global (e.g., to World Wildlife Fund) and local levels? How do they share nonmarket information and strategies among their subsidiaries and private sector and governmental partners? We also need more research on how social movements use new social media technologies in their CSR efforts and how these efforts affect MNEs. As noted by Gerbaudo (2012), these new platforms have played a major role in recent social movement campaigns, based on both generating publicity and/or improving the organization and coordination of protests and other aspects of activism. More broadly, we need to deepen our understanding of these internet-based movements (Shirky, 2011), including terrorist organizations, ransomware, and efforts to pressure companies to do or not do business with certain partners. This will prove highly useful to researchers interested in determining whether social media strategies are effective in changing MNE CSR practices.

The global pandemic underscores the interconnectedness among issues and actors across jurisdictions as a complex, multilevel, multi-actor problem (Lawton, Dorobantu, Rajwani, & Sun, 2020). It unfolded at global, national, and local levels, as well as within specific industry sectors and global supply chains. It has exposed and reinforced interdependencies among countries, sectors (business, government, civil society), groups, and individuals, while also exacerbating divisions and differences in the form of economic and medical nationalism at the global and national level. As just one example, of the more than 175 vaccine development efforts underway, including 33 in human trials, many involve cross-national, cross-sectoral cooperation, including the Pfizer and BioNTech vaccine, the Oxford-Astra Zeneca vaccine, and the GeoVax/BravoVax vaccine (Calloway, 2020). Another area that naturally involves multiple sectors and actors and at multiple levels is the incorporation of the United Nations Sustainable Development Goals (UNSDGs) as an organizing framework for CSR-/sustainability-related activities. Although developed for global scale action, one of the UNSDGs deals specifically with partnerships to implement the goals, and the goals themselves have been adopted by not just governments but by NGOs and MNEs, often as part of cross-sectoral collaborations (Van Tulder, Rodrigues, Mirza, & Sexsmith, 2021; van Zanten & van Tulder, 2018).

From a methodological vantage, political scientists and sociologists have begun to use social network analysis to explore social movements. For example, Saunders (2007) builds on Diani’s definition of a social movement to document the linkages between different types of environmental organizations in the UK. Isa and Himelboim (2018) applied a social networks approach to examine patterns of information flow within the #FreeAJStaff movement on Twitter, collecting 22 months of data, resulting in social networks created by 71,326 users and 149,650 social ties (mentions and replies) among them. These tools, including spatial methods, such as geographic information systems, could be leveraged to “map” the complex interactions between MNEs, social movements actors, and other governmental and nongovernmental stakeholders.

### Conclusion: Maintaining Relevance and Rigor in Multinational NMS

Early IB scholars were deeply engaged in broad questions about the efficacy and potential shortcomings of economic globalization; however, most IB scholarship over the past two decades has not been as present in debates about international business policy and practice, effectively ceding that space to political scientists and economists. The introduction of the *Journal of International Business Policy* reflects a useful response to this shortcoming, as does the work of some individual IB scholars (Contractor, 2021). Yet, questions regarding the relevant role and influence of global institutions – what political scientists and management scholars deem “global governance” – have not been of primary interest to IB scholars (Doh et al., 2015). The nonmarket environment encapsulates a wide range of phenomenon and practices, ranging from broad external challenges to MNEs, such as terrorism, populism, natural disasters, and managing energy transitions, to more endogenous concerns, such as training executives for positions involving nonmarket issues, understanding cultural differences related to differences in governmental practices, and structuring global cybersecurity apparatus. These topics have not always been viewed as central to IB as a field.

In response, we believe multinational NMS research can serve as a helpful conduit for addressing phenomena that cross sectors (business, government, and civil society), occur at multiple levels of analysis (global, national, local, organizational, individual), involve multiple types of issues (commercial, social, economic), and span disciplinary fields (international political economy, management studies). We encourage ambitious efforts to address these broad questions through the application of innovative theoretical perspectives, the use of rigorous and appropriate methodological tools, and continued attention to real world phenomena.

## Notes

1. An additional term, international business diplomacy (also referred to as “business diplomacy” and “corporate diplomacy”), has appeared in some practitioner- and policy-oriented literature; however, it does not yet appear with much frequency in the scholarly multinational nonmarket strategy literature, although, as we note in the final section, may offer some future research opportunities by linking with and integrating international relations with IB scholarship.
2. In our review, we do not include studies that treat “corruption” as a contextual condition in the same way as national culture and institutional voids that firms respond to through market strategies like entry mode and subsidiary ownership choice. We do, however, include articles that examine how firms address corruption through nonmarket actions and the instances when MNEs themselves engage in corrupt activities. For more details, see our subsection “Dealing with corruption” under the heading of “Integration of CPA and SCSR”.
3. For instance, lobbying has been one of the most widely studied corporate political action initiatives in the US context. However, the keyword search in our database results in only 11 papers on cross-border corporate lobbying (e.g., Barron, et al., 2017; Kim, 2019; Weil, 2018). On the other hand, we suspect that a significant number of multinational CPA articles (especially those that explore political ties and MNE–host government relationships) do cover MNE “lobbying” activities, but they simply do not use this terminology. With respect to another popular political tactic, campaign contributions, the current literature does not suggest that this is a major area of interest. The only two papers that touch upon this issue in our database are Calluzzo, Dong, & Godsell (2017) and Shi, Gao, & Aguilera (2021). The former studies the investment of foreign sovereign wealth funds (SWFs) in US-based firms. SWFs are found to be attracted to firms engaged in US campaign finance, and that firm campaign contributions increase after SWF investment. The latter finds that increasing ownership stakes held by foreign institutional investors leads the focal US-headquartered companies to engage in more campaign contributions. We believe the relative paucity of MNE NMS research on campaign contributions may be a function of the relative lack of availability of data in many jurisdictions that do not have a reporting expectation comparable to the United States requirements under the Federal Election Campaign Act, and enforced by the Federal Election Commission.
4. An understudied area concerns intellectual property rights protection in host countries and technology transfer from established MNEs to local firms (Spencer, 2008). More research is needed to provide deeper insights into how MNEs accommodate governments’ demand for technology without losing their key knowledge assets (Prud'homme & von Zedtwitz, 2019; Sun, Deng, & Wright, 2021).
5. For instance, the model predicts that a weak MNE from a strong home country will have more bargaining power than a strong MNE from a weak home country. We are unaware of empirical attempts to evaluate this conjecture.

## Electronic supplementary material

Below is the link to the electronic supplementary material.

Supplementary material 1 (DOCX 82 kb)
