# Supplementary material for: Navigating cross-border institutional complexity: A review and assessment of multinational nonmarket strategy research
Source: J Int Bus Stud. 2021 Jun 21;52(9):1818–53. doi: 10.1057/s41267-021-00438-x (PMC8215093; doi:10.1057/s41267-021-00438-x)
Supplement: Supplementary file 1 — Supplementary material 1 (DOCX 82 kb) [file 41267_2021_438_MOESM1_ESM.docx]

**APPENDIX**

**ADDITIONAL TABLES**

**Table A Highly Cited Pre-2000 Publications on Multinational NMS**

| **AUTHORS** | **TITLE** | **JOURNAL** | **YEAR** | **CITES** |
| --- | --- | --- | --- | --- |
| Boddewyn, J. J., & Brewer, T.L. | International-business political-behavior: new theoretical directions | *Academy of Management Review*, 19(1): 119-143. | 1994 | 426 |
| Kobrin, S. J. | Political risk: a review and reconsideration | *Journal of International Business Studies*, 10(1): 67-80. | 1979 | 246 |
| Murtha, T. P., & Lenway, S. A. | Country capabilities and the strategic state: how national political institutions affect multinational corporations’ strategies | *Strategic Management Journal*, 15: SI (Summer): 113-129. | 1994 | 240 |
| Schuler, D. A. | Corporate political strategy and foreign competition: The case of the steel industry | *Academy of Management Journal*, 39(3): 720-727. | 1996 | 182 |
| Hillman, A. J., & Keim, G | International variation in the business-government interface: institutional and organizational considerations | *Academy of Management Review*, 20(1): 193-214. | 1995 | 165 |
| Rugman, A. M., & Verbeke, A. | Corporate strategies and international environmental policy | *Journal of International Business Studies*, 29(4): 819-834. | 1998 | 106 |
| Robock, S. H. | Political risk: identification and assessment | *Columbia Journal of World Business*, 6(4): 6-20. | 1971 | 103 |
| Ring, P. S., Lenway, S. A., & Govekar, M. | Management of the political imperative in international business | *Strategic Management Journal*, 11(2): 141-151. | 1990 | 93 |
| Lenway, S.A, & Murtha, T. P. | The state as strategist in international-business research | *Journal of International Business Studies*, 25(3): 513-535. | 1994 | 87 |
| Brouthers, K.D., & Bamossy, G. J. | The role of key stakeholders in international joint venture negotiations: case studies from Eastern Europe | *Journal of International Business Studies*, 28(2): 285-308. | 1997 | 80 |
| Butler, K. C., & Joaquin, D. C. | A note on political risk and the required return on foreign direct investment | *Journal of International Business Studies*, 29 (3): 599-607. | 1998 | 82 |
| Simon, J. D. | A theoretical perspective on political risk | *Journal of International Business Studies*, 15 (3): 123-143. | 1984 | 71 |
| Fitzpatrick, M. | The definition and assessment of political risk in international business: A review of the literature | *Academy of Management Review*, 8 (2): 248-254. | 1983 | 70 |
| Brewer, T. L. | An issue-area approach to the analysis of MNE-government relations | *Journal of International Business Studies*, 23 (2): 295-309. | 1992 | 64 |

Note: The web of science citation data shown in the table was downloaded on February 15, 2021.

**Table B Evolution of Research on Multinational NMS by Journal Type**

|  | **General Management Journals** | **IB Specialist Journals** | **Nonmarket Strategy Journals** | **Journals from other Disciplines** | **Total** |
| --- | --- | --- | --- | --- | --- |
| 2000 | 1 | 0 | 0 | 0 | 1 |
| 2001 | 1 | 4 | 3 | 0 | 8 |
| 2002 | 1 | 2 | 1 | 0 | 4 |
| 2003 | 1 | 3 | 2 | 0 | 6 |
| 2004 | 1 | 5 | 0 | 0 | 6 |
| 2005 | 3 | 4 | 1 | 0 | 8 |
| 2006 | 4 | 6 | 2 | 1 | 13 |
| 2007 | 1 | 1 | 7 | 0 | 9 |
| 2008 | 2 | 4 | 4 | 0 | 10 |
| 2009 | 4 | 3 | 2 | 0 | 9 |
| 2010 | 1 | 14 | 5 | 0 | 20 |
| 2011 | 1 | 6 | 3 | 0 | 10 |
| 2012 | 2 | 10 | 11 | 0 | 23 |
| 2013 | 3 | 8 | 1 | 1 | 13 |
| 2014 | 1 | 15 | 6 | 1 | 23 |
| 2015 | 2 | 21 | 7 | 3 | 33 |
| 2016 | 4 | 21 | 8 | 3 | 36 |
| 2017 | 5 | 17 | 0 | 2 | 24 |
| 2018 | 3 | 31 | 6 | 1 | 41 |
| 2019 | 5 | 22 | 0 | 1 | 28 |
| 2020 | 12 | 22 | 8 | 0 | 42 |
| **Total (%)** | **58 (15.8)** | **219 (59.7)** | **77 (21.0)** | **13 (3.5)** | **367 (100)** |

Note: The row of “2020” contains 29 articles formally published in 2020 and 13 articles that had been online in press by December 2020 and would be formally published from January 2021 onwards.

**Table C Article Distribution across Academic Journals and Major Themes**

|  | CPA | SCSR | CPA & SCSR | Total number (%) |
| --- | --- | --- | --- | --- |
| *General Management Journals* |  |  |  |  |
| Academy of Management Journal | 4 | 6 | 0 | 10 |
| Academy of Management Review | 1 | 1 | 0 | 2 |
| Administrative Science Quarterly | 2 | 1 | 0 | 3 |
| Journal of Management | 2 | 0 | 0 | 2 |
| Journal of Management Studies | 1 | 4 | 1 | 6 |
| Management Science | 0 | 0 | 1 | 1 |
| Organization Science | 0 | 1 | 0 | 1 |
| Organization Studies | 1 | 0 | 0 | 1 |
| Strategic Management Journal | 18 | 7 | 7 | 32 |
| *Subtotal* | ***29*** | ***20*** | ***9*** | ***58 (15.8)*** |
|  |  |  |  |  |
| *IB Specialist Journals* |  |  |  |  |
| Global Strategy Journal | 15 | 3 | 2 | 20 |
| International Business Review | 18 | 15 | 1 | 34 |
| Journal of International Business Studies | 39 | 25 | 8 | 72 (19.6) |
| Journal of International Management | 8 | 2 | 2 | 12 |
| Journal of World Business | 24 | 16 | 10 | 50 |
| Management International Review | 19 | 6 | 6 | 31 |
| *Subtotal* | ***123*** | ***67*** | ***29*** | ***219 (59.7)*** |
|  |  |  |  |  |
| *Nonmarket Strategy Journals* |  |  |  |  |
| Business Ethics Quarterly | 0 | 5 | 0 | 5 |
| Business & Politics | 5 | 5 | 4 | 14 |
| Business & Society | 8 | 9 | 4 | 21 |
| Journal of Business Ethics | 1 | 29 | 7 | 37 |
| *Subtotal* | ***14*** | ***48*** | ***15*** | ***77 (21.0)*** |
|  |  |  |  |  |
| *Journals from Other Disciplines* |  |  |  |  |
| American Journal of Political Science | 2 | 0 | 0 | 2 |
| American Sociological Review | 1 | 0 | 0 | 1 |
| International Marketing Review | 0 | 5 | 0 | 5 |
| Journal of Corporate Finance | 2 | 0 | 0 | 2 |
| Journal of International Marketing | 1 | 1 | 0 | 2 |
| Review of Economics and Statistics | 1 | 0 | 0 | 1 |
| *Subtotal* | ***7*** | ***6*** | ***0*** | ***13 (3.5)*** |
|  |  |  |  |  |
| Total number (%) | ***173 (47.1)*** | ***141 (38.4)*** | ***53 (14.4)*** | **367 (100)** |

**Table D Article Distribution across Academic Journals and Research Methodology**

|  | Conceptual | Empirical | | | Total number (%) |
| --- | --- | --- | --- | --- | --- |
|  |  | Quantitative | Qualitative | Mixed |  |
| *General Management Journals* |  |  |  |  |  |
| Academy of Management Journal | 0 | 10 | 0 | 0 | 10 |
| Academy of Management Review | 2 | 0 | 0 | 0 | 2 |
| Administrative Science Quarterly | 0 | 3 | 0 | 0 | 3 |
| Journal of Management | 1 | 1 | 0 | 0 | 2 |
| Journal of Management Studies | 0 | 4 | 2 | 0 | 6 |
| Management Science | 0 | 1 | 0 | 0 | 1 |
| Organization Science | 0 | 1 | 0 | 0 | 1 |
| Organizational Studies | 0 | 0 | 1 | 0 | 1 |
| Strategic Management Journal | 1 | 26 | 5 | 0 | 32 |
| *Subtotal* | ***4*** | ***46*** | ***8*** | ***0*** | ***58 (15.8)*** |
|  |  |  |  |  |  |
| *IB Specialist Journals* |  |  |  |  |  |
| Global Strategy Journal | 7 | 8 | 5 | 0 | 20 |
| International Business Review | 6 | 22 | 6 | 0 | 34 |
| Journal of International Business Studies | 11 | 54 | 6 | 1 | 72 (19.6) |
| Journal of International Management | 2 | 5 | 5 | 0 | 12 |
| Journal of World Business | 13 | 15 | 21 | 1 | 50 |
| Management International Review | 3 | 20 | 7 | 1 | 31 |
| *Subtotal* | ***42*** | ***124*** | ***50*** | ***3*** | ***219 (59.7)*** |
|  |  |  |  |  |  |
| *Nonmarket Strategy Journals* |  |  |  |  |  |
| Business Ethics Quarterly | 2 | 3 | 0 | 0 | 5 |
| Business & Politics | 5 | 1 | 8 | 0 | 14 |
| Business & Society | 4 | 13 | 4 | 0 | 21 |
| Journal of Business Ethics | 6 | 11 | 19 | 1 | 37 |
| *Subtotal* | ***17*** | ***28*** | ***31*** | ***1*** | ***77 (21.0)*** |
|  |  |  |  |  |  |
| *Journals from Other Disciplines* |  |  |  |  |  |
| American Journal of Political Science | 0 | 2 | 0 | 0 | 2 |
| American Sociological Review | 0 | 0 | 1 | 0 | 1 |
| International Marketing Review | 0 | 0 | 5 | 0 | 5 |
| Journal of Corporate Finance | 0 | 2 | 0 | 0 | 2 |
| Journal of International Marketing | 0 | 2 | 0 | 0 | 2 |
| Review of Economics and Statistics | 0 | 1 | 0 | 0 | 1 |
| *Subtotal* | **0** | **7** | **6** | **0** | ***13 (3.5)*** |
|  |  |  |  |  |  |
| Total number (%) | ***63 (17.2)*** | ***205 (55.9)*** | ***95 (25.9)*** | ***4 (1.1)*** | **367 (100)** |

**Table E Article Distribution across Country/Region Identity**

**in Host/Home Country Categories**

|  | Home Country | Host Country | Total |
| --- | --- | --- | --- |
| China | 41 | 45 | **86** |
| United States | 55 | 17 | **72** |
| Western Europe | 46 | 15 | **61** |
| Latin America | 9 | 26 | **35** |
| Africa | 5 | 29 | **34** |
| India | 6 | 17 | **23** |
| Japan | 14 | 0 | **14** |
| Eastern Europe and Russia | 5 | 10 | **15** |
| South Korea | 4 | 5 | **9** |

Note: Some conceptual and empirical articles are not amenable to this home-host classification. Also, research articles do not necessarily specify full lists of the individual countries/regions in their samples. For example, a paper can just state that the sample firms are from 20 host developing countries without providing a full list of these countries. The countries/regions shown in the table are those explicitly mentioned by the research articles. For simplicity, we group individual Western European countries and Latin American countries to two broad categories respectively. Information about the breakdown within a region is available upon request.

**Table F Highly Cited Articles on International CPA Ranked by Web of Science Citations**

|  | **AUTHORS & Year** | **Article** | **TOPIC(S)** | **TYPE** | **THEORY** | **TOTAL CITES** | **CITES/YR** |
| --- | --- | --- | --- | --- | --- | --- | --- |
| 1 | Delios & Henisz (2003) | Political hazards, experience, and sequential entry strategies: the international expansion of Japanese firms, 1980–1998. *Strategic Management Journal*, 24(11): 1153–1164 | Political risk; Foreign entry strategy | Empirical (Quantitative) | The stages model of Internationalization | 378 | 21 |
| 2 | Hitt, Bierman, Uhlenbruck, & Shimizu (2006) | The importance of resources in the internationalization of professional service firms: the good, the bad, and the ugly. *Academy of Management Journal*, 49(6): 1137–1157 | Firm internationalization | Empirical (Quantitative) | RBV | 358 | 23.9 |
| 3 | Cui & Jiang (2012) | State ownership effect on firms' FDI ownership decisions under institutional pressure: a study of Chinese outward-investing firms. *Journal of International Business Studies*, 43(3): 264-284 | SOE internationalization | Empirical (Quantitative) | Institutional theory and RDT | 319 | 35.4 |
| 4 | Holburn & Zelner (2010) | Political capabilities, policy risk, and international investment strategy: evidence from the global electric power generation industry. *Strategic Management Journal*, 31(12): 1290-1315 | Political risk; Location choice | Empirical (Quantitative) | RBV and TCE | 297 | 27 |
| 5 | Wang, Hong, Kafouros, & Wright (2012) | Exploring the role of government involvement in outward FDI from emerging economies. *Journal of International Business Studies*, 43(7): 655-676 | SOE internationalization | Empirical (Quantitative) | Resource-based and institutional perspectives | 271 | 30.1 |
| 6 | Hillman & Wan (2005) | The determinants of MNE subsidiaries' political strategies: evidence of institutional duality. *Journal of International Business Studies*, 36(3): 322–340 | Subsidiary-level political strategies | Empirical (Quantitative) | Institutional theory | 211 | 13.3 |
| 7 | Mezias (2002) | Identifying liabilities of foreignness and strategies to minimize their effects: the case of labor lawsuit judgments in the United States. *Strategic Management Journal*, 23(3): 229–244 | Managing liabilities of foreignness | Empirical (Quantitative) | FDI/Liability of foreignness literature | 211 | 11.1 |
| 8 | Frynas, Mellahi, & Pigman (2006) | First mover advantages in international business and firm-specific political resources. *Strategic Management Journal*, 27(4): 321-345 | MNE-host government relation | Empirical (Qualitative) | RDT and RBV | 209 | 13.9 |
| 9 | Siegel (2007) | Contingent political capital and international alliances: Evidence from South Korea. *Administrative Science Quarterly*, 52(4): 621-666 | Political ties | Empirical (Quantitative) | Social network theory | 194 | 14.9 |
| 10 | Cuervo-Cazurra, Inkpen, Musacchio, & Ramaswamy (2014) | Governments as owners: state-owned multinational companies. *Journal of International Business Studies*, 45(8): 919-942 | SOE internationalization | Conceptual (Special issue editorial) | Agency theory, TCE, RBV, RDT, and institutional theory | 187 | 26.7 |
| 11 | Henisz & Zelner (2005) | Legitimacy, interest group pressures, and change in emergent institutions: the case of foreign investors and host country governments. *Academy of Management Review*, 30(2): 361-382 | MNE-host government relation/bargaining | Conceptual | Institutional theory | 187 | 11.7 |
| 12 | García-Canal & Guillén (2008) | Risk and the strategy of foreign location choice in regulated industries. *Strategic Management Journal*, 29(10): 1097-1115 | Political risk management and location choice | Empirical (Quantitative) | Corporate political strategy literature; internationalization theories | 183 | 14.1 |
| 13 | Sun, Mellahi, & Thun (2010) | The dynamic value of MNE political embeddedness: the case of the Chinese automobile industry. *Journal of International Business Studies*, 41(7): 1161-1182 | MNE-host government relation; IJV | Empirical (Qualitative) | RDT and social embeddedness theory | 168 | 15.3 |
| 14 | Li, Zhou, & Shao (2009) | Competitive position, managerial ties, and profitability of foreign firms in China: an interactive perspective. *Journal of International Business Studies*, 40(2): 339-352 | Political ties | Empirical (Quantitative) | Competitive strategy literature and social network theory | 168 | 14 |
| 15 | Meyer, Ding, Li, & Zhang (2014) | Overcoming distrust: how state-owned enterprises adapt their foreign entries to institutional pressures abroad. *Journal of International Business Studies*, 45(8): 1005–1028 | SOE internationalization | Empirical (Quantitative) | Institutional theory | 166 | 23.7 |
| 16 | Ramamurti (2001) | The obsolescing 'bargaining model'? MNC-Host developing country relations revisited. *Journal of International Business Studies*, 32(1): 23-39 | MNE-Host Government Relation | Conceptual | MNE-host government bargaining model | 157 | 7.9 |
| 17 | Luo (2001) | Toward a cooperative view of MNC-host government relations: building blocks and performance implications. *Journal of International Business Studies*, 32(3): 401-419 | MNE-Host Government Relation | Empirical (Quantitative) | MNE-host government bargaining model | 147 | 7.4 |
| 18 | Zhang, Zhou, & Ebbers (2011) | Completion of Chinese overseas acquisitions: institutional perspectives and evidence. *International Business Review*, 20(2): 226-238 | Cross-border M&A | Empirical (Quantitative) | Institution-based view | 123 | 12.3 |
| 19 | Xia, Ma, Lu, & Yiu (2014) | Outward foreign direct investment by emerging market firms: a resource dependence logic. *Strategic Management Journal*, 35(9): 1343-1363 | Emerging market firm internationalization | Empirical (Quantitative) | RDT | 119 | 17 |
| 20 | Li, Cui, & Lu (2014) | Varieties in state capitalism: outward FDI strategies of central and local state-owned enterprises from emerging economy countries. *Journal of International Business Studies*, 45(8): 980-1004 | SOE internationalization | Conceptual | Institutional theory | 107 | 15.3 |
| 21 | Feinberg & Gupta (2009) | MNC subsidiaries and country risk: Internalization as a safeguard against weak external institutions. *Academy of Management Journal*, 52(2): 381-399 | Political risk | Empirical (Quantitative) | TCE | 102 | 8.5 |
| 22 | Liang, Ren, & Sun (2015) | An anatomy of state control in the globalization of state-owned enterprises. *Journal of International Business Studies*, 46(2): 223-240 | SOE internationalization | Empirical (Quantitative) | Agency theory | 91 | 15.2 |
| 23 | Duanmu (2014) | State-owned MNCs and host country expropriation risk: The role of home state soft power and economic gunboat diplomacy. *Journal of International Business Studies*, 45(8), 1044-1060 | SOE internationalization; Bilateral political relations/diplomacy | Empirical (Quantitative) | TCE | 89 | 12.7 |
| 24 | Doh, Teegen, & Mudambi (2004) | Balancing private and state ownership in emerging markets' telecommunications infrastructure: country, industry, and firm influences. *Journal of International Business Studies*, 35(3): 233–250 | MNE-host government bargaining | Empirical (Quantitative) | TCE | 76 | 4.5 |
| 25 | Du & Boateng (2015) | State ownership, institutional effects and value creation in cross-border mergers & acquisitions by Chinese firms. *International Business Review*, 24(3): 430-442 | SOE internationalization; Cross-border M&A | Empirical (Quantitative) | Institutional theory | 75 | 12.5 |
| 26 | Estrin, Meyer, Nielsen, & Nielsen (2016) | Home country institutions and the internationalization of state owned enterprises: A cross-country analysis. *Journal of World Business*, 51(2): 294-307 | SOE internationalization | Empirical (Quantitative) | New institutional economics | 73 | 14.6 |
| 27 | Brockman, Rui, & Zou (2013) | Institutions and the performance of politically connected M&As. *Journal of International Business Studies*, 44(8): 833-852 | Political ties | Empirical (Quantitative) | Agency theory | 71 | 8.9 |
| 28 | Blumentritt & Nigh (2002) | The integration of subsidiary political activities in multinational corporations. *Journal of International Business Studies*, 33(1): 57-77 | Headquarter subsidiary relations | Empirical (Quantitative) | RDT | 71 | 3.7 |

Note: The citation data were collected from the Web of Sciences in mid-February 2021.

**Table G Highly Cited Articles on International SCSR Ranked by Web of Science Citations**

|  | **AUTHORS & Year** | **ARTICLE** | **TOPIC(S)** | **Type** | **THEORY(IES)** | **TOTAL CITES** | **CITES/YR** |
| --- | --- | --- | --- | --- | --- | --- | --- |
| 1 | Maignan & Ralston (2002) | Corporate social responsibility in Europe and the U.S.: insights from businesses’ self-presentations. *Journal of International Business Studies*, 33(3): 497–514 | Comparative CSR | Empirical (Quantitative) | Strategic CSR literature | 613 | 32.3 |
| 2 | Christmann & Taylor (2001) | Globalization and the environment: determinants of firm self-regulation in China. *Journal of International Business Studies*, 32(3): 439-458 | Environmental issues | Empirical (Quantitative) | Environmental regulation literature | 532 | 26.6 |
| 3 | King, Lenox, & Terlaak (2005) | The strategic use of decentralized institutions: exploring certification with the ISO 14001 management standard. *Academy of Management Journal*, 48(6): 1091-1106 | Environmental issues; ISO certification | Empirical (Quantitative) | Institutional theory and TCE | 440 | 27.5 |
| 4 | Christmann (2004) | Multinational companies and the natural environment: determinants of global environmental policy. *Academy of Management Journal*, 47(5): 747-760 | MNEs’ global environmental policy | Empirical (Quantitative) | Stakeholder theory | 380 | 22.4 |
| 5 | Jackson & Apostolakou (2010) | Corporate social responsibility in Western Europe: An institutional mirror or substitute? *Journal of Business Ethics*, 94(3): 371-394 | Determinants of CSR practices | Empirical (Quantitative) | Neo-institutional theory and comparative institutional analysis | 353 | 32.1 |
| 6 | Gardberg & Fombrun (2006) | Corporate citizenship: creating intangible assets across institutional environments. *Academy of Management Review*, 31(2): 329-346 | Corporate citizenship (strategic CSR) | Conceptual | Institutional theory; Theory of strategic balance | 351 | 23.4 |
| 7 | Husted & Allen (2006) | Corporate social responsibility in the multinational enterprise: strategic and institutional approaches. *Journal of International Business Studies*, 37(6): 838-849 | Organizational CSR | Empirical (Quantitative) | Institutional theory | 347 | 23.1 |
| 8 | Christmann & Taylor (2006) | Firm self-regulation through international certifiable standards: determinants of symbolic versus substantive implementation. *Journal of International Business Studies*, 37(6): 863-878 | ISO certification | Empirical (Quantitative) | TCE | 337 | 22.5 |
| 9 | Teegen, Doh, & Vachani (2004) | The importance of nongovernmental organizations (NGOs) in global governance and value creation: an international business research agenda. *Journal of International Business Studies*, 35(6): 463-483 | NGO | Conceptual  (Perspective paper) | NA | 318 | 18.7 |
| 10 | Strike, Gao, & Bansa (2006) | Being good while being bad: social responsibility and the international diversification of US firms. *Journal of International Business Studies*, 37(6): 850-862 | CSR/CSiR | Empirical (Quantitative) | RBV | 305 | 20.3 |
| 11 | Child & Tsai (2005) | The dynamic between firms’ environmental strategies and institutional constraints in emerging economies: Evidence from China and Taiwan. *Journal of Management Studies*, 42(1): 95-125 | Environment and institutions | Empirical (Qualitative) | Institutional theory | 245 | 15.3 |
| 12 | Campbell, Eden, & Miller (2012) | Multinationals and corporate social responsibility in host countries: Does distance matter? *Journal of International Business Studies*, 43(1): 84-106 | MNEs and CSR in host countries | Empirical (Quantitative) | Liability of foreignness literature | 202 | 22.4 |
| 13 | Kolk (2016) | The social responsibility of international business: From ethics and the environment to CSR and sustainable development. *Journal of World Business*, 51(1): 23-34 | Environmental issues; sustainable development | Conceptual  (Review paper) | NA | 181 | 36.2 |
| 14 | Perego & Kolk (2012) | Multinationals' accountability on sustainability: the evolution of third-party assurance of sustainability reports. *Journal of Business Ethics*, 110(2): 173–190 | CSR reporting | Empirical (Quantitative) | Social and environmental accounting literature; Institutional theory | 169 | 18.8 |
| 15 | Yang & Rivers (2009) | Antecedents of CSR practices in MNCs' subsidiaries: a stakeholder and institutional perspective. *Journal of Business Ethics*, 86(2, supplement): 155–169 | Antecedents of strategic CSR; Stakeholder management | Conceptual | Stakeholder theory, institutional theory | 154 | 12.8 |
| 16 | Kolk (2010) | Trajectories of sustainability reporting by MNCs. *Journal of World Business*, 45(4): 367-374 | CSR reporting | Empirical (Qualitative) | Strategic CSR literature | 141 | 12.8 |
| 17 | Surroca, Tribo, & Zahra (2013) | Stakeholder pressure on MNEs and the transfer of socially irresponsible practices to subsidiaries. *Academy of Management Journal*, 56(2): 549-572 | CSiR | Empirical (Quantitative) | Institutional theory | 137 | 17.1 |
| 18 | Kolk & Pinkse (2008) | A perspective on multinational enterprises and climate change: Learning from "an inconvenient truth"? *Journal of International Business Studies*, 39(8): 1359–1378 | Climate change | Conceptual | RBV | 121 | 9.3 |
| 19 | Marano, Tashman, & Kostova (2017) | Escaping the iron cage: liabilities of origin and CSR reporting of emerging market multinational enterprises. *Journal of International Business Studies*, 48(3): 386-408 | CSR reporting | Empirical (Quantitative) | Institutional theory | 114 | 28.5 |
| 20 | Marano & Kostova (2016) | Unpacking the institutional complexity in adoption of CSR practices in multinational enterprises. *Journal of Management Studies*, 53(1): 28-54 | CSR practice adoption | Empirical (Quantitative) | Institutional theory | 95 | 19 |
| 21 | Keig, Brouthers, & Marshall (2015) | Formal and informal corruption environments and multinational enterprise social irresponsibility. *Journal of Management Studies*, 52(1): 89-116. | CSiR | Empirical (Quantitative) | Institutional theory | 95 | 15.8 |
| 22 | Zyglidopoulos (2002) | The social and environmental responsibilities of multinationals: Evidence from the Brent Spar case. *Journal of Business Ethics*, 36(1-2): 141-151 | NGO; Stakeholder management | Empirical (Qualitative) | Stakeholder theory | 90 | 4.7 |
| 23 | Gifford, Kestler, & Anand (2010) | Building local legitimacy into corporate social responsibility: Gold mining firms in developing nations. *Journal of World Business*, 45(3): 304-311 | Host country CSR projects | Empirical (Qualitative) | Institutional theory | 82 | 7.5 |
| 24 | Escobar & Vredenburg (2011) | Multinational oil companies and the adoption of sustainable development: a resource-based and institutional theory interpretation of adoption heterogeneity. *Journal of Business Ethics*, 98(1): 39–65 | Sustainable development | Empirical (Qualitative) | Institutional theory, RBV | 78 | 7.8 |
| 25 | Martín-Tapia, Aragón-Correa, & Rueda-Manzanares (2010) | Environmental strategy and exports in medium, small and micro-enterprises. *Journal of World Business*, 45(3): 266-275 | Environmental issues | Empirical (Quantitative) | RBV | 77 | 7 |
| 26 | Crilly (2011) | Predicting stakeholder orientation in the multinational enterprise: A mid-range theory. *Journal of International Business Studies*, 42(5): 694-717 | Stakeholder management | Empirical (Qualitative) | RDT and stakeholder theory | 75 | 7.5 |
| 27 | Madsen (2009) | Does corporate investment drive a “race to the bottom” in environmental protection? a reexamination of the effect of environmental regulation on investment. *Academy of Management Journal*, 52(6): 1297-1318 | Environmental issues | Empirical (Quantitative) | Regulatory competition model; internationalization process theory | 75 | 6.3 |
| 28 | Arthaud-Day (2005) | Transnational corporate social responsibility: A Tri-dimensional approach to international CSR research. *Business Ethics Quarterly*, 15(1): 1-22 | MNEs and global CSR activities | Conceptual | NA | 75 | 4.7 |
| 29 | Park & Ghauri (2015) | Determinants influencing CSR practices in small and medium sized MNE subsidiaries: A stakeholder perspective. *Journal of World Business*, 50(1): 192-204 | Determinants of subsidiary CSR practices | Empirical (Quantitative) | Stakeholder theory | 74 | 12.3 |
| 30 | Brammer, Pavelin, & Porter (2009) | Corporate charitable giving, multinational companies and countries of concern. *Journal of Management Studies*, 46(4): 575-596 | Corporate philanthropy | Empirical (Quantitative) | Stakeholder theory | 73 | 6.1 |
| 31 | Pinkse & Kolk (2012) | Multinational enterprises and climate change: Exploring institutional failures and embeddedness. *Journal of International Business Studies*, 43(3): 332-341 | Climate change | Conceptual | Institutional theory | 70 | 7.8 |

Note: The citation data were collected from the Web of Sciences in mid-February 2021.

**Table H Highly Cited Articles on CPA & SCSR Ranked by Web of Science Citations**

|  | **AUTHORS** | **TITLE** | **JOURNAL & YEAR** | **TOPIC(S)** | **Type** | **THEORY(IES)** | **TOTAL CITES** | **CITES/YR** |
| --- | --- | --- | --- | --- | --- | --- | --- | --- |
| 1 | Doh & Guay (2006) | Corporate social responsibility, public policy, and NGO activism in Europe and the United States: an institutional-stakeholder perspective. *Journal of Management Studies*, 43(1): 47-73 | | NGO and public policy | Empirical (Qualitative) | Institutional theory; stakeholder theory | 505 | 33.7 |
| 2 | Rodriguez, Siegel, Hillman, & Eden (2006) | Three lenses on the multinational enterprise: politics, corruption, and corporate social responsibility. *Journal of International Business Studies*, 37(6): 733-746 | | CPA, CSR and Corruption (Special issue introduction) | Conceptual | NA | 241 | 16.1 |
| 3 | Luo (2006) | Political behavior, social responsibility, and perceived corruption: a structuration perspective. *Journal of International Business Studies*, 37(6): 747-766 | | CPA, CSR and Corruption | Empirical (Quantitative) | Giddens' theory of structuration | 133 | 8.9 |
| 4 | Spencer & Gomez (2011) | MNEs and corruption: the impact of national institutions and subsidiary strategy. *Strategic Management Journal*, 32(3): 280-300 | | Bribery/corruption in host countries | Empirical (Quantitative) | Institutional theory | 99 | 9.9 |
| 5 | Cuervo-Cazurra (2008) | The effectiveness of laws against bribery abroad. *Journal of International Business Studies*, 39(4): 634–651 | | bribery in host countries | Empirical (Quantitative) | New Institutional Economics | 95 | 7.3 |
| 6 | Montiel, Husted, & Christmann (2012) | Using private management standard certification to reduce information asymmetries in corrupt environments. *Strategic Management Journal*, 33(9): 1103-1113 | | Corruption; environmental issues | Empirical (Quantitative) | New institutional economics | 92 | 10.2 |
| 7 | Doh, Rodrigues, Saka-Helmhout, & Makhija (2017) | International business responses to institutional voids. *Journal of International Business Studies*, 48(3): 293-307 | | CPA and CSR | Conceptual (Special issue editorial) | NA | 87 | 21.8 |
| 8 | Stevens, Xie, & Peng (2016) | Toward a legitimacy-based view of political risk: The case of Google and Yahoo in China. *Strategic Management Journal*, 37(5): 945-963 | | Political risk | Empirical (Qualitative) | Institutional theory | 84 | 16.8 |
| 9 | Cuervo-Cazurra (2016) | Corruption in international business. *Journal of World Business*, 51(1): 35-49 | | Corruption | Conceptual | Agency theory, TCE, RBV, RDT, and Institutional theory | 84 | 16.8 |
| 10 | Detomasi (2008) | The political roots of corporate social responsibility. *Journal of Business Ethics*, 82(4): 807-819 | | Political determinants of strategic CSR | Conceptual | NA | 79 | 6.1 |
| 11 | Reimann, Ehrgott, Kaufmann, & Carter (2012) | Local stakeholders and local legitimacy: MNEs' social strategies in emerging economies. *Journal of International Management*, 18(1): 1-17 | | Stakeholder management | Empirical (Quantitative) | Stakeholder theory | 75 | 8.3 |
| 12 | Boddewyn & Doh (2011) | Global strategy and the collaboration of MNEs, NGOs, and governments for the provisioning of collective goods in emerging markets. *Global Strategy Journal*, 1(2): 345-361 | | Collaboration of MNEs, NGOs, and host governments to provide collective goods in emerging markets | Conceptual | TCE and Institutional theory | 72 | 7.2 |
| 13 | Jamali & Mirshak (2010) | Business-conflict linkages: revisiting MNCs, CSR, and conflict. *Journal of Business Ethics*, 93(3): 443–464 | | MNE sociopolitical engagement strategies in conflict areas | Empirical (Qualitative) | Stakeholder management and CPA literatures | 59 | 5.4 |
| 14 | Oetzel & Getz (2012) | Why and how might firms respond strategically to violent conflict? *Journal of International Business Studies*, 43(2): 166-186 | | Violent conflicts | Empirical (Quantitative) | Stakeholder theory | 57 | 6.3 |
| 15 | Beddewela & Fairbrass (2016) | Seeking legitimacy through CSR: Institutional pressures and corporate responses of Multinationals in Sri Lanka. *Journal of Business Ethics*, 136(3): 503-522 | | Strategic CSR to enhance legitimacy and develop political relationships in host economies | Empirical (Qualitative) | Institutional theory | 55 | 11 |
| 16 | Darendeli & Hill (2016) | Uncovering the complex relationships between political risk and MNE firm legitimacy: insights from Libya. *Journal of International Business Studies*, 47(1): 68-92. | | CSR activities as a hedge against political risk/hazard | Empirical (Qualitative) | Institutional theory | 54 | 10.8 |

Note: The citation data were collected from the Web of Sciences in mid-February 2021.

**Table I Key Contributors to Multinational Nonmarket Strategy Literature since 2000**

| **Number of articles** | | | **Number of articles weighted by co-authors** | | | **Citations (Google Scholar)** | | | **Author impact score** | | |
| --- | --- | --- | --- | --- | --- | --- | --- | --- | --- | --- | --- |
| **1** | Doh JP | 12 | **1** | Kolk A | 4.76 | **1** | Doh JP | 3159 | **1** | Christmann P | 1877 |
| **2** | Kolk A | 9 | **2** | Doh JP | 4.50 | **2** | Christmann P | 3025 | **2** | Kolk A | 1709 |
| **3** | Puck JF | 7 | **3** | Luo Y | 4.17 | **3** | Kolk A | 2377 | **3** | Doh JP | 1317 |
| **4** | Husted BW | 6 | **4** | Husted BW | 2.40 | **4** | Husted BW | 1624 | **4** | Luo Y | 899 |
|  | Lawton TC | 6 | **5** | Puck JF | 2.33 | **5** | Hillman AJ | 1399 | **5** | Husted BW | 764 |
|  | Mohr AT | 6 | **6** | Boddewyn JJ | 2.27 | **6** | Hensiz WJ | 1201 | **6** | Hillman AJ | 695 |
| **7** | Boddweyn JJ | 5 |  | Christmann P | 2.27 | **7** | Teegen HJ | 1114 | **7** | Henisz WJ | 601 |
|  | Christmann P | 5 |  | Cuervo-Cazzuro A | 2.27 | **8** | Vachani S | 971 | **8** | Cuervo-Cazzuro A | 567 |
|  | Cuervo-Cazzuro A | 5 | **9** | Lawton TC | 2.25 | **9** | Luo Y | 922 | **9** | Siegel JI | 466 |
|  | Luo Y | 5 | **10** | Bucheli M | 2.00 | **10** | Cuervo-Cazzuro A | 886 | **10** | Jamali DR | 454 |
|  | Meyer KE | 5 |  | Oetzel JM | 2.00 | **11** | Cui L | 809 | **11** | Delios AK | 399 |
|  | Rajwani TS | 5 | **12** | Blumentritt TP | 1.80 | **12** | Delios AK | 797 | **12** | Teegen HJ | 371 |
| **13** | Bucheli M | 4 |  | Hillman AJ | 1.80 | **13** | Mellahi K | 779 | **13** | Cui L | 365 |
|  | Cui, L | 4 | **14** | Marano V | 1.78 | **14** | Jamali DR | 600 | **14** | Vachani S | 324 |
|  | Hillman AJ | 4 | **15** | Park Byung I | 1.60 | **15** | Meyer KE | 599 | **15** | Zyglidopoulos SC | 273 |
|  | Li Jing | 4 |  | Rajwani TS | 1.60 | **16** | Li Jing | 537 | **16** | Blumentritt TP | 267 |
|  | Ma X | 4 | **17** | Meyer KE | 1.56 |  | Marano V | 537 | **17** | Mellahi K | 258 |
|  | Marano V | 4 | **18** | Oh CH | 1.50 | **18** | Siegel JI | 530 | **18** | Yang X | 206 |
|  | Newenham-Kahindi A | 4 | **19** | Duanmu J-L | 1.45 | **19** | Wright M | 528 | **19** | Boddewyn JJ | 204 |
|  | Oh CH | 4 |  | Jamali DR | 1.45 | **20** | Kostova T | 473 | **20** | Kostova T | 195 |
|  | Park Byung I | 4 |  |  |  |  |  |  |  |  |  |
|  | White GO | 4 |  |  |  |  |  |  |  |  |  |
|  |  |  |  |  |  |  |  |  |  |  |  |
|  |  |  |  |  |  |  |  |  |  |  |  |
|  |  |  |  |  |  |  |  |  |  |  |  |

Note: Author impact score is calculated as citations to papers within the database, weighted by the number of co-authors on each paper. Citations were extracted from Google Scholar as of February 15, 2021.
